# Detection of Δ9-Tetrahydrocannabinol Impairment Using Resting-State Functional Near-Infrared Spectroscopy: A Randomized Clinical Trial

**DOI:** 10.1001/jamanetworkopen.2025.56647

**Published:** 2026-01-30

**Authors:** Moshe Berchansky, A. Eden Evins, Bryn Evohr, Zachary Himmelsbach, Gladys N. Pachas, Keerthana Deepti Karunakaran, Bracha Laufer Goldshtein, Nisan Ozana, Jodi M. Gilman

**Affiliations:** 1 Faculty of Engineering, Institute of Nanotechnology and Advanced Materials, The Gonda Multidisciplinary Brain Research Center, Bar-Ilan University, Ramat Gan, Israel; 2 Center for Addiction Medicine, Department of Psychiatry, Massachusetts General Hospital, Harvard Medical School, Boston; 3 MGH/HST Athinoula A. Martinos Center for Biomedical Imaging, Department of Radiology, Massachusetts General Hospital, Harvard Medical School, Boston; 4 School of Computer and Electrical Engineering, Tel-Aviv University, Tel Aviv, Israel

## Abstract

**Importance:**

The primary psychoactive compound in cannabis, ∆9-tetrahydrocannabinol (THC) induces intoxication and functional impairment, raising safety concerns in driving. The traditional impairment detection method, behavioral field sobriety tests (FSTs), are subject to bias.

**Objective:**

To determine whether resting-state functional near-infrared spectroscopy (fNIRS) can detect THC-related impairment with greater accuracy and a lower rate of false positives than FSTs.

**Design, Setting, and Participants:**

This double-blind, randomized, crossover trial was conducted from January 2017 to January 2021 at a single site. Eligible participants were adults aged 18 to 55 years who used cannabis. Analyses were performed from November 2024 to November 2025.

**Interventions:**

Participants received a single oral dose of synthetic THC (range, 5-80 mg) intended to induce intoxication or placebo in separate visits.

**Main Outcome and Measures:**

fNIRS scans were acquired before and approximately 100 and 200 minutes after study drug administration to assess prefrontal cortex responses at rest and during a working memory task. Machine learning models trained on fNIRS data were then used to identify clinically determined THC-induced impairment. The primary outcome of this study was accuracy of THC-induced impairment classification using fNIRS data as compared with an FST. Model performance was quantified using false-positive rate, precision, recall, F1 score, and area under the receiver operating curve (ROC-AUC).

**Results:**

A total of 183 participants (mean [SD] age, 25.3 [6.3] years; 90 [49.2%] female) who used cannabis for a median (IQR) of 6.5 (4-7) days per week completed at least 1 study visit. fNIRS data collected during rest produced a classifier for impairment, with an ROC-AUC of 0.87 (95% CI, 0.83 to 0.91), accuracy of 0.90 (95% CI, 0.88 to 0.92), and false-positive rate of 0.05 (95% CI, 0.04 to 0.07), using clinical impairment assessment as ground truth. The FST showed an ROC-AUC of 0.75 (95% CI, 0.74 to 0.76), accuracy of 0.69 (95% CI, 0.67 to 0.71), and a false-positive rate of 0.34 (95% CI, 0.32 to 0.36). fNIRS performed significantly better than the FST in precision (difference = 0.23; 95% CI, 0.14 to 0.33; *P* < .001), accuracy (difference = 0.15; 95% CI, 0.10 to 0.19; *P* < .001), false-positive rate (difference = −0.25, 95% CI, −0.31 to −0.20; *P* < .001), and ROC-AUC (difference = 0.08; 95% CI, 0.01 to 0.14; *P* = .005).

**Conclusions and Relevance:**

In this crossover trial of THC vs placebo, THC intoxication produced prefrontal cortex activation patterns detectable with resting state fNIRS neuroimaging, producing a neural signature of THC-induced impairment that was superior to FSTs for individual-level impairment identification. These findings lay the groundwork for further exploration of fNIRS as a tool for detecting impairment.

**Trial Registration:**

ClinicalTrials.gov Identifier: NCT03655717

## Key Points

**Question:**

Can a portable neuroimaging method (functional near-infrared spectroscopy [fNIRS]) improve detection of impairment due to ∆9-tetrahydrocannabinol (THC)–induced intoxication over current benchmark behavioral assessments?

**Findings:**

In this crossover trial of 183 adults who received doses of THC and/or placebo, 6-minute fNIRS measurements of resting prefrontal cortex activity identified THC impairment with superior accuracy compared with 45-minute expanded field sobriety tests and with comparable accuracy to fNIRS measurements conducted during attention task performance.

**Meaning:**

These findings suggest that portable fNIRS neuroimaging provides more objective, accurate, and rapid detection of THC-induced impairment than conventional field sobriety methods, offering significant advantages for cannabis impairment detection in roadside, workplace, and research settings.

## Teaser

This crossover trial compares the accuracy of resting-state functional near-infrared spectroscopy vs standard field sobriety testing to detect ∆9-tetrahydrocannabinol (THC) impairment among adults who use cannabis.

## Introduction

Cannabis intoxication, caused by ∆9-tetrahydrocannabinol (THC), negatively impacts cognitive and psychomotor performance.^1^ The prevalence of significant blood THC concentrations in injured motor vehicle drivers more than doubled after commercialization of cannabis markets,^2^ and THC is now frequently detected in injured drivers.^3^ Accurate assessment of impairment due to THC intoxication has become a pressing public safety challenge.^2,4^ In contrast with blood alcohol concentration, which reliably correlates with impairment, no THC or THC metabolite concentration threshold in blood, saliva, or urine accurately estimates functional deficits observed during THC intoxication.^5^ Because of individual differences in tolerance, metabolism, and neurocognitive adaptation, impairment classification based solely on THC presence or concentration are inherently unreliable.^5,6^ THC metabolite concentration thresholds are additionally problematic as indicators for impairment because THC metabolites can remain detectable in body fluids for weeks of abstinence, long after intoxication has resolved.^7,8^ Field sobriety tests (FSTs)^9^ that were developed to detect impairment due to alcohol intoxication have poor sensitivity to THC-specific deficits^6^ and high rates of false-positive ratings and operator bias.^10,11^ These limitations underscore an urgent need for an objective method to accurately detect THC impairment and distinguish THC exposure with functional impairment from THC exposure without impairment.^6,12^

The effect of THC on cognitive function is thought to arise at least in part from binding to cannabinoid 1 receptors concentrated in the fronto-limbic brain circuit.^13^ Cannabinoid 1 receptor activation in the ventral tegmental area stimulates dopamine release, modulating γ-aminobutyric acid and glutamate signaling,^14^ leading to decreased functional connectivity within the mesocorticolimbic circuit and increased functional connectivity in the prefrontal cortex.^15,16,17^ THC exposure also activates reward-related brain regions, including the medial prefrontal cortex,^18^ and enhances blood flow in these areas.^19^ THC intoxication increases oxygenated hemoglobin responses detected with functional near infrared spectroscopy (fNIRS) in the prefrontal cortex during n-back working memory task performance,^20,21^ and individuals experiencing THC intoxication exhibit stronger oxygenated hemoglobin responses in the prefrontal cortex than those receiving similar doses of THC who demonstrated few or no clinical signs of intoxication.^20^ Task-based prefrontal cortical fNIRS data have been shown to reliably distinguish individuals who are impaired due to THC intoxication from adults who were not impaired following THC dosing,^20^ suggesting that prefrontal cortical activity changes detectable with fNIRS could serve as an objective indicator of THC intoxication.

Building on additional findings that THC intoxication disrupts prefrontal cortex activity,^22^ here we aimed to discover the accuracy of individual THC-impairment classification based solely on fNIRS-acquired measures of prefrontal resting-state brain activity. Here we tested an artificial intelligence framework that utilizes brain activity patterns to distinguish scans conducted when participants were clinically impaired from THC intoxication from scans conducted when participants were not clinically impaired. We hypothesized that THC-induced intoxication could be reliably identified using fNIRS-measured prefrontal cortex activity patterns during resting state as well as during cognitive task performance. Specifically, we hypothesized that fNIRS data would provide objective neural signatures that correlate with clinically assessed impairment, surpassing traditional behavioral impairment detection methods.

## Methods

### Study Design and Participants

Study procedures for this crossover trial were approved by the Partners Human Subjects Committee. All participants gave written informed consent prior to study activities and were compensated for completing study procedures. Reporting follows the Consolidated Standards of Reporting Trials (CONSORT) reporting guideline. Recruitment and data acquisition were conducted from January 2017 through January 2021 at Massachusetts General Hospital. Participants, aged 18 to 55 years, reported using cannabis at least weekly.^20,21^ Sex, race, and ethnicity were assessed by participants’ response to a fixed-category question. Race categories included American Indian or Alaska Native, Asian, Black or African American, White, more than 1 race, and unknown, while ethnicity categories included Hispanic or Latino, not Hispanic or Latino, and unknown; race and ethnicity were included to characterize the study sample and to assess potential differences in outcomes across demographic groups. THC metabolite 11-Nor-Δ9-tetrahydrocannabinol-9-carboxylic acid (THC-COOH) concentration greater than 50 ng/mL using liquid chromatography-tandem mass spectrometry, with levels normalized to creatinine concentration, at screening confirmed recent cannabis use.^8^ Participants were asked to abstain from intoxicating substance use in the mornings prior to study visits. The trial protocol and statistical analysis plan can be found in Supplement 1.

### Interventions

The data were acquired during a double-blind, crossover study in which participants received a single, personalized dose of synthetic THC (dronabinol) and identical placebo capsules on separate study visits, randomized for order, at least 1 week apart. Dronabinol dose was individualized with the goal of producing intoxication at a dose that was well tolerated by each participant, up to a maximum of 80 mg. The dose was determined through clinical interview ascertaining usual cannabis consumption patterns, level of intoxication, and adverse effects in each participant, with some consideration for sex and body mass index (eMethods in Supplement 2).

#### Study Procedures

**Figure 1.**
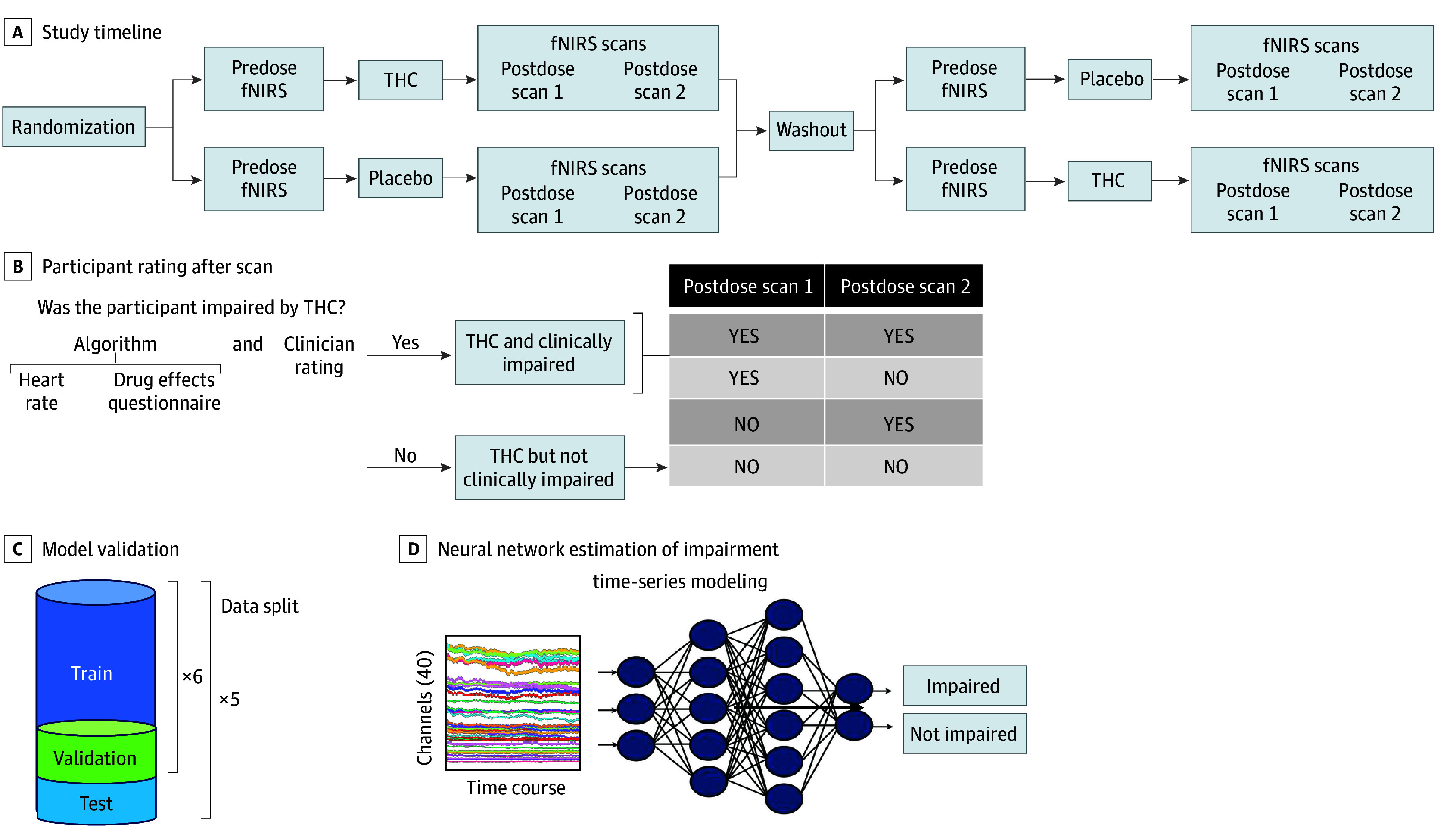
Schematic Overview of Study Design, Data Partitioning, and Time-Series Modeling This Figure illustrates the workflow of the study from data acquisition through model prediction. A, Each participant completed functional near-infrared spectroscopy (fNIRS) scans before and after receiving either Δ9-tetrahydrocannabinol (THC) or placebo. Scans were performed at 3 time points: predose, postdose 1, and postdose 2. A subset of participants also received a third postdose scan. B, Based on a composite clinical assessment, including heart rate, self-reported intoxication, and clinician ratings, participants were labeled as clinically impaired or not clinically impaired at each postdose scan. C, The dataset was divided into 5 test folds, with each training set further split into 6 subsets for model validation. D, Input fNIRS time-series data were fed into a neural network model to predict the impairment.

Prefrontal hemodynamics were evaluated using a 20-channel fNIRS system during n-back task performance and 6 minutes of resting state before and at approximately 100 and 200 minutes after taking the study drug (Figure 1A). The n-back involved a 6-minute session comprising alternating 30-second blocks of the 0-back and 2-back conditions of the letter n-back working memory task.^21^ Heart rate (beats per minute) and subjective intoxication levels on the Drug Effects Questionnaire (DEQ; 5 items rated from 0 [no effect] to 100 [maximum effect])^23^ were measured before and approximately every 20 minutes for 240 minutes after THC or placebo administration.

#### Field Sobriety Testing

Certified drug recognition experts, who were members of the study staff, performed a structured extended FST as outlined in the Advanced Roadside Impaired Driving Enforcement manual,^9^ approximately 120 minutes after study drug administration. The extended FST included horizontal gaze nystagmus, pupillary response, walk-and-turn, and 1-leg stand with balance and counting phases, conducted in a standardized sequence.

### fNIRS Acquisition

Cortical hemodynamic data were collected using a continuous-wave fNIRS system (NIRSport2 [NIRx Inc]) with 8 sources emitting light at 760 and 850 nm wavelengths and 8 photodetectors. The placement followed the International 10-20 System,^24^ with the central probe aligned with the Fpz (frontopolar midline) site. fNIRS data were preprocessed using a standard pipeline,^25^ including principal component, analysis-based motion correction, filtering, and conversion from optical density to oxygenated and deoxygenated hemoglobin concentration measures. The input data to the impairment classification models were oxyhemoglobin and deoxyhemoglobin time series from 20 channels per participant.

### Impairment Definition

As described previously,^20^ without a standard, accepted definition for impairment, we developed a 2-step process^20^ to determine impairment due to THC intoxication. Participants were considered impaired at the time of a fNIRS scan if (1) two clinical raters independently rated the participant as impaired, using all available data except fNIRS data, and (2) an algorithm combining physiological and psychological data (heart rate and self-reported intoxication [DEQ]) classified the participant as impaired following study drug administration (eMethods in Supplement 2). Scans conducted in participants when rated as impaired by both methods served as ground truth for impairment models (Figure 1B). Among participants who became impaired, some were impaired at both postdose scans, and some were inconsistently impaired (ie, impaired at one postdose scan but not the other).

### Model Design and Tuning

For the purpose of model training, nonimpaired scans comprised 3 types: (1) postplacebo scans, (2) predose scans, and (3) post-THC scans without clear clinical impairment. This resulted in fewer impaired scans (112 for n-back and 111 for resting) than nonimpaired scans (800 for n-back and 782 for resting). To optimize impairment classification, we compared several models, including Granite TTM R2^26^ and the Detach Rocket^27^ architecture, and a parallel extraction feature technique involving a combination of 4 submodels (eFigure 1 in Supplement 2). Input data were passed into each model for feature extraction. We used cross-entropy loss for all models, aside from Detach Rocket, where we used ridge regularization. Both loss terms were used to train the impairment classification models. Each model included additional data processing steps (eMethods in Supplement 2). For model calibration and prediction, we adopted a nested cross-validation framework. The full dataset was divided into 5 testing folds. For each of these outer folds, the remaining 4 folds of data were subdivided into 6 smaller validation folds (*k* models) (Figure 1C). For each validation fold, a model was trained on the other 5 inner folds, then calibrated and scored on the validation fold. Models were independently trained and calibrated using isotonic regression to map model outputs to well-calibrated probability estimates in the 0 to 1 range. Each data point in the outer test fold was classified based on the average outcome from all *k* models. The resulting features were combined and passed into a final module that projected the features into a class prediction score (ranging from 0 for not impaired to 1 for impaired) (Figure 1D). To generate binary predictions at the scan level, we computed the F1 score across all possible decision thresholds on the validation set and selected the threshold that yielded the highest F1 score. This optimal threshold was then applied to classify each scan in the outer test fold as either impaired or not impaired. This ensemble-style approach reduces the influence of any single model’s variance and improves overall predictive reliability. Participant-level data partitioning was strictly enforced; all scans from a given individual were assigned to a single fold to prevent data leakage and ensure independence across training, validation, and test sets.

As a robustness check, we also trained separate machine learning models on data that excluded scans from the THC sessions without clinical impairment. To fit this model, we excluded participants who were not clinically impaired during either postdose scan from the training and validation datasets, assigning them exclusively to the test set. We used this approach to assess model generalizability and robustness in identifying THC-induced impairment without influence from participants who were never clinically impaired by THC.

### Statistical Analysis

Analyses were conducted using Python version 3.12.9 (Python Software Foundation) and R version 4.4.0 (R Project for Statistical Computing) from November 2024 to November 2025. The primary outcome of this study was accuracy of THC-induced impairment classification using fNIRS data, as assessed by machine learning models trained on prefrontal hemodynamic features. Classification performance was evaluated against a clinical ground truth of impairment defined as both clinician-rated impairment and impairment based on physiological measures and self-reported intoxication at the time of the fNIRS scan. Model performance was quantified using standard metrics, including false-positive rate, precision (the ratio of true positives to the sum of true positives and false positives), recall (the ratio of true positives to the sum of true positives and false negatives), F1 score (the harmonic mean of precision and recall), and area under the receiver operating curve (ROC-AUC). We used multilevel models, accounting for within-subject dependence, to test whether probability scores, heart rate, or DEQ scores differed when clinically impaired vs not clinically impaired post dose, as well as between postdose and predose scans. Subgroup analyses were conducted to assess model performance by participant age, sex, race and ethnicity, and cannabis use frequency.

#### Bootstrap Comparison of Classifier Performance

To quantify statistical uncertainty in classifier performance and formally compare the fNIRS-based classifier with the FST-based classifier, we implemented a paired bootstrap hypothesis testing procedure. All comparisons were performed on the shared test-set scans for which both models produced predictions, ensuring a one-to-one matched evaluation.

For each bootstrap iteration, we resampled test-set indices with replacement, using stratified resampling to preserve the original proportion of impaired and not impaired cases. For each bootstrap sample, we computed the following performance metrics for both classifiers: ROC-AUC, accuracy, precision, recall, F1 score, and false-positive rate. We then computed the paired difference between the fNIRS classifier and the FST classifier on each metric.

This procedure was repeated 1000 times, yielding an empirical sampling distribution of paired differences for each performance metric. From these distributions, we calculated 95% CIs, SEs, and bootstrap *P* values, defined as the proportion of bootstrap differences less than or equal to 0. Bootstrap analyses were conducted separately for the resting-state and n-back models. A metric was considered statistically significant when the 95% CI did not include zero and the *P* value was less than .05. All reported results reflect this bootstrap-based uncertainty estimation and hypothesis testing framework.

#### Sample Size

Given the novelty of using fNIRS to detect THC-induced impairment, we aimed to maximize enrollment within budget constraints to evaluate the performance of fNIRS-based models within resource limitations. Power analyses were not conducted.

## Results

**Figure 2.**
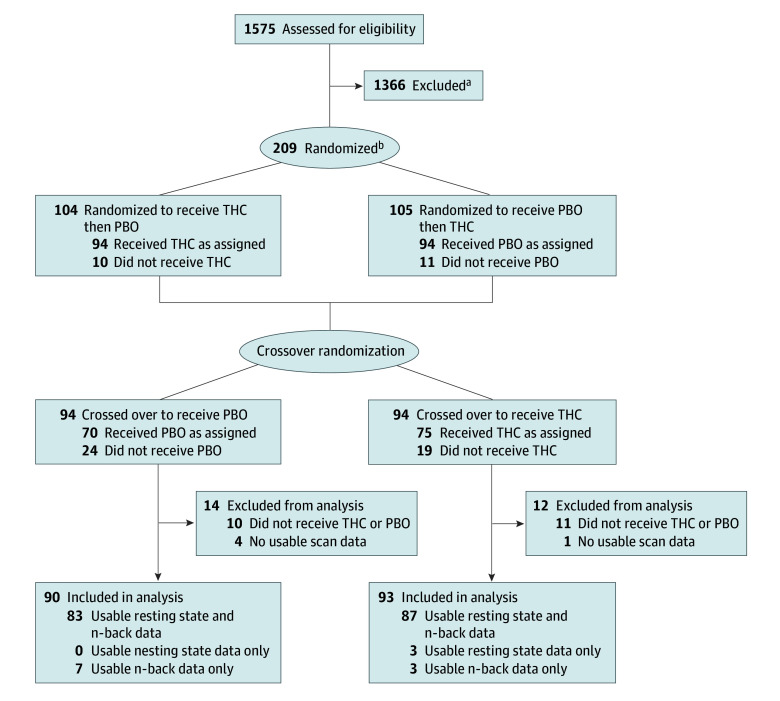
Flow Diagram ^a^Excluded individuals were not eligible or chose not to participate. Specific reasons for exclusion were not recorded. ^b^Participants were randomly assigned for order to receive both dronabinol, a synthetic form of Δ9-tetrahydrocannabinol (THC), and placebo (PBO).

A total of 183 participants completed at least 1 study visit. Participants’ mean (SD) age was 25.3 (6.3) years. Of all participants, 90 (49.2%) were female, 12 (6.6%) were Asian, 21 (11.5%) were Black or African American, and 126 (68.9%) were White. Participants used cannabis for a median (IQR) of 6.5 (4-7) days per week (eTable 1 in Supplement 2). fNIRS scans were of sufficient quality to analyze in 173 participants during resting state and 180 participants during n-back task performance (Figure 2). The mean (SD) interval between study visits was 21.6 (33.6) days. Following THC administration, 84 resting-state scans and 89 n-back scans were conducted when participants were clinically impaired, and 71 resting state scans and 73 n-back scans were conducted when participants were considered to be not clearly clinically impaired (eTable 2 in Supplement 2). The mean (SD) study drug dose of THC was 35.0 (11.7) mg in the group who met clinical impairment criteria and 37.7 (17.0) mg in those who did not meet clinical impairment criteria (*P* = .25) (eTable 3 in Supplement 2).

### Model Performance

Resting-state fNIRS features yielded a classifier with a precision of 0.60 (95% CI, 0.53-0.69; SE, 0.041), recall of 0.56 (95% CI, 0.47-0.65; SE = 0.046), and an F1 score of 0.58 (95% CI, 0.51-0.65; SE = 0.038). Accuracy was 0.90 (95% CI, 0.88-0.92; SE = 0.008), the false-positive rate was 0.05 (95% CI, 0.04-0.07; SE, 0.007), and the ROC-AUC was 0.87 (95% CI, 0.83-0.91; SE = 0.019) (Table 1).

**Table 1.** Classifier Performance Across fNIRS Resting-State, n-Back Task, and Field Sobriety Evaluation for Detecting THC Impairment^a^

Metric	Observed value (95% CI)	Standard error
Resting		
Precision	0.60 (0.53-0.69)	0.041
Recall	0.56 (0.47-0.65)	0.046
F1 score	0.58 (0.51-0.65)	0.038
Accuracy	0.90 (0.88-0.92)	0.008
False-positive rate	0.05 (0.04-0.07)	0.007
ROC-AUC	0.87 (0.83-0.91)	0.019
n-Back		
Precision	0.55 (0.48-0.63)	0.039
Recall	0.61 (0.51-0.69)	0.047
F1 score	0.58 (0.50-0.65)	0.037
Accuracy	0.89 (0.86-0.91)	0.010
False-positive rate	0.07 (0.05-0.09)	0.009
ROC-AUC	0.85 (0.80-0.89)	0.022
Field sobriety evaluation		
Precision	0.36 (0.34-0.37)	0.008
Recall	0.84 (0.80-0.86)	0.016
F1 score	0.50 (0.48-0.52)	0.010
Accuracy	0.69 (0.67-0.71)	0.008
False-positive rate	0.34 (0.32-0.36)	0.010
ROC-AUC	0.75 (0.73-0.76)	0.009

Abbreviations: fNIRS, functional near-infrared spectroscopy; ROC-AUC, area under the receiver operating curve; THC, Δ9-tetrahydrocannabinol.

^a^ Performance metrics with bootstrap-derived 95% CIs and standard errors for fNIRS resting-state, n-back, and field sobriety evaluations in detecting THC impairment.

Similar results were demonstrated for the n-back task, with an ROC-AUC of 0.85 (95% CI, 0.80-0.89; SE = 0.022), accuracy of 0.89 (95% CI, 0.86-0.91; SE = 0.010), and with a false-positive rate of 0.07 (95% CI, 0.05-0.09; SE = 0.009). Precision was 0.55 (95% CI, 0.48-0.63; SE = 0.039), and recall was 0.61 (95% CI, 0.51-0.69; SE = 0.047), yielding an F1 score of 0.58 (95% CI, 0.50-0.65; SE = 0.037) (Table 1). Model performance on the resting state and n-back was not significantly different (eTable 4 in Supplement 2).

The FST showed a precision of 0.36 (95% CI, 0.34-0.37), accuracy of 0.69 (95% CI, 0.67-0.71;), a false-positive rate of 0.34 (95% CI, 0.32-0.36), and ROC-AUC of 0.75 (95% CI, 0.73-0.76) (Table 1). Paired bootstrap analysis demonstrated that the fNIRS-based classifier significantly outperformed the FST-based classifier across nearly all performance metrics (Table 2).

**Table 2.** Statistical Comparison of fNIRS-Based Impairment Detection and Field Sobriety Testing^a^

Metric	Observed difference (95% CI)	Standard error	*P* value^b^
Resting			
Precision	0.23 (0.14 to 0.33)	0.049	<.001
Recall	−0.30 (−0.42 to −0.18)	0.062	<.001
F1 score	0.05 (−0.03 to 0.14)	0.047	.13
Accuracy	0.15 (0.10 to 0.19)	0.024	<.001
False-positive rate	−0.25 (−0.31 to −0.20)	0.027	<.001
ROC-AUC	0.08 (0.01 to 0.14)	0.031	.005
n-Back			
Precision	0.25 (0.17 to 0.35)	0.046	<.001
Recall	−0.22 (−0.34 to −0.09)	0.062	<.001
F1 score	0.10 (0.02 to 0.19)	0.045	.009
Accuracy	0.15 (0.10 to 0.20)	0.025	<.001
False-positive rate	−0.24 (−0.30 to −0.19)	0.028	<.001
ROC-AUC	0.07 (0.01 to 0.14)	0.034	.02

Abbreviations: fNIRS, functional near-infrared spectroscopy; ROC-AUC, area under the receiver operating curve.

^a^ Observed differences in performance metrics between fNIRS resting-state and n-back machine learning models and the field sobriety (certified drug recognition experts [DRE]) evaluation, with 95% CIs and standard errors derived from 1000 bootstrap resamples. Positive values indicate higher values of the fNIRS model relative to the DRE (precision, F1, accuracy, and ROC-AUC), while negative values indicate higher values in the DRE models (false-positive rate and recall).

^b^ *P* values reflect paired bootstrap-based significance testing.

Resting-state fNIRS showed significantly better performance compared with the FST in precision (difference = 0.23; 95% CI, 0.14 to 0.33; *P* < .001), accuracy (difference = 0.15; 95% CI, 0.10 to 0.19; *P* < .001), false-positive rate (difference = −0.25; 95% CI, −0.31 to −0.20; *P* < .001); and ROC-AUC (difference = 0.08; 95% CI, 0.01 to 0.14; *P* = .005). The F1 score difference was not statistically significant (difference = 0.05; 95% CI, −0.03 to 0.14; *P* = .13), due to recall being higher for the drug recognition expert, as expected (difference = −0.30; 95% CI, −0.42 to −0.18; *P* < .001). The n-back classifier was also superior to the FST in all metrics except recall (Table 2).

**Figure 3.**
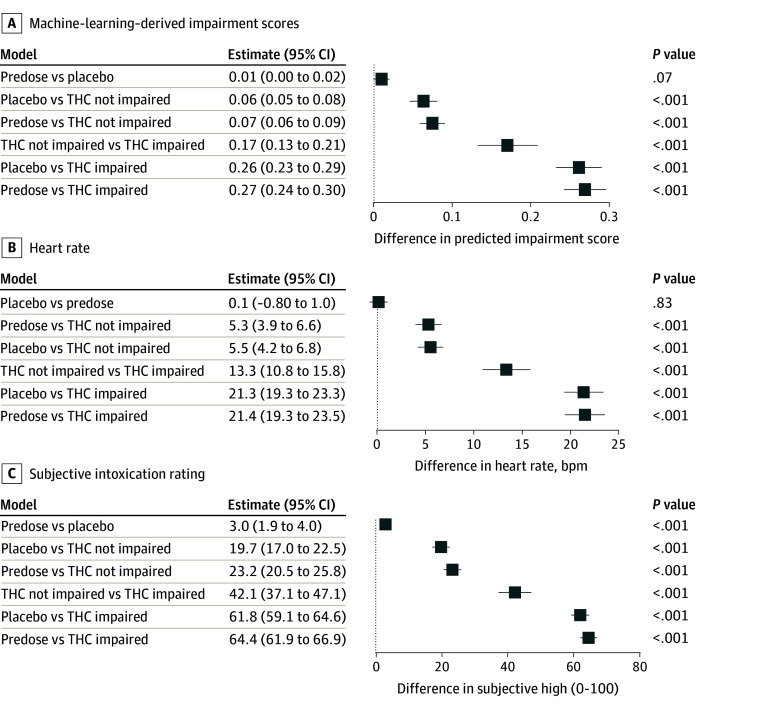
Group Comparisons of Impairment Scores, Heart Rate, and Drug Effects Questionnaire (DEQ) Ratings Across Dosing Conditions Model-estimated contrasts are shown for machine learning-derived impairment scores (A), heart rate (beats per minute [bpm]; B), and DEQ subjective intoxication ratings (C). Panels depict model-estimated mean differences and 95% CIs for key contrasts (comparing predose, placebo, Δ9-tetrahydrocannabinol [THC]–impaired, and not THC-impaired conditions). All estimates were derived from multilevel models accounting for within-participant dependence.

As expected, for each participant, the model classified predose and placebo scans with the lowest impairment scores (Figure 3A). Impairment prediction scores (Figure 3A), as well as heart rate changes (Figure 3B) and DEQ ratings (Figure 3C), were significantly higher in the THC-impaired condition compared with all other conditions. Also as expected, no significant differences in these measures were observed between the placebo and predose conditions. See eFigure 2 in Supplement 2 for visual plots of impairment scores, heart rate changes, and subjective intoxication ratings per condition. Within-individual performance of the prediction model during each scan is depicted in eFigure 3 in Supplement 2.

### Temporal Specificity

To examine whether the neural patterns underlying impairment classification differences aligned temporally with clinical assessment of impairment, we evaluated model performance on the subset of participants who were rated as clinically impaired at one but not both post-THC scans. In the subsample of 52 participants who were clinically impaired at one but not both scan times, temporal analyses showed that the resting-state model identified impairment with a precision of 0.63 (95% CI, 0.50-0.76), recall of 0.48 (95% CI, 0.35-0.62), an F1 score of 0.54 (95% CI, 0.42-0.65), accuracy of 0.85 (95% CI, 0.82-0.89), false-positive rate of 0.06 (95% CI, 0.03-0.10), and ROC-AUC of 0.84 (95% CI, 0.77-0.90) (eTable 5 in Supplement 2). These findings indicate that the model retained discriminative ability even when impairment status changed across time within individuals. This performance was similar to that reported for the full sample, indicating that the onset and offset of the neural patterns indicating impairment from intoxication align with the onset and offset of clinical signs and symptoms of impairment.

### Alternative Models and Sensitivity Checks

The alternative models, Granite TTM-R2 and Granite Combined, yielded similar performance (eTable 6 in Supplement 2). Overall, the models demonstrated robust performance in predicting impairment probability across age, sex, race, and cannabis use frequency (eFigure 4 in Supplement 2).

### Channel Importance

In addition to evaluating overall model performance, we analyzed the contribution of individual channels to classification ability. We used the mean ROC-AUC scores for each oxygenated fNIRS channel over the validation set, using the Detach Rocket model, with each channel assessed independently. The analyzed brain regions include the left and right medial dorsal and ventral prefrontal cortex and the medial prefrontal cortex. Our findings indicate that the mid-upper channels, located in the dorsomedial region, contributed less to model performance, possibly due to poorer signal quality resulting from hair interference (eFigure 5 in Supplement 2).

## Discussion

In this crossover trial, we tested an individualized, functional, data-driven approach for detecting impairment due to THC intoxication and demonstrated strong performance metrics using machine learning models trained on fNIRS features, which were significantly more accurate than expanded FSTs. fNIRS features indicative of impairment had high accuracy, low false-positive rates, and aligned temporally with changes in clinical signs of impairment during study visits, thereby increasing confidence that prefrontal fNIRS measures are detecting neural signatures of cannabis impairment consistent with impairment in the moment. The approach utilized time series models to enhance accuracy while maintaining computational efficiency.

The activation patterns of the prefrontal cortex identified in this study align with existing literature on cognitive deficits during cannabis intoxication. Placebo-controlled studies consistently demonstrate that dose-dependent deficits in faculties related to driving, such as attention,^28^ psychomotor function,^29^ impulse control,^17^ and decision-making,^30^ are associated with acute cannabis intoxication. These cognitive domains are predominantly supported by the prefrontal cortex, a region essential for higher-order executive functions, including working memory and the temporary storage and manipulation of information necessary for complex cognitive tasks.^31^ These neural signatures may act as reliable biomarkers of impairment, providing a neurobiological method to complement subjective, cognitive, and biochemical evaluations of THC intoxication.

We note that there was no significant advantage to using the n-back compared with the resting state scans; the strong classifier performance using resting-state data suggests that THC-induced impairment may not solely reflect task-related cognitive alterations but rather broader disruptions in prefrontal network dynamics. The ability to classify impairment using resting state fNIRS data may broaden practical applications of this approach because task-based assessments can be time-consuming and require active participant engagement, which may introduce biases. Brain-based assessments of cannabis-induced impairment, such as portable fNIRS, may complement cognitive approaches to THC impairment detection (eg, DRUID mobile app)^32^ because resting-state fNIRS data assessed during intoxication accurately identified impaired states without needing nonintoxicated baseline information.

Integration of advanced machine learning models represents a significant advancement in applying portable neuroimaging technologies to real-world challenges. This study benefits from a rigorous double-blind, randomized crossover approach. The false-positive rate was low and occurred most commonly following THC dosed to cause intoxication in participants who did not meet our clinical criteria for impairment at the time of the scan. It is thus not known whether these apparent false positive classifications represent inaccuracy in our clinical classification of impairment or false positive impairment classification based on fNIRS data.

### Limitations

This study has limitations. Further work is needed to establish the generalizability of these findings to THC consumption methods with different pharmacokinetics (eg, inhalation) and to polysubstance intoxication. Further work is also needed to establish whether the neural signature identified in this study, which was temporally specific to clinical cannabis intoxication, can discriminate cannabis intoxication from other impairing conditions, such as alcohol intoxication or sleep deprivation. Additionally, although THC doses were individually tailored to achieve intoxication, more than one-half of participants did not meet clinical impairment criteria following THC administration; this likely reflects the high tolerance typical of regular cannabis users, in whom substantial neuroadaptations can attenuate both subjective intoxication and observable behavioral changes despite significant THC exposure. As a result, the neural impairment patterns detected by fNIRS may underestimate the magnitude of THC-related effects in less tolerant populations. An additional limitation of this study is that plasma THC and metabolite concentrations were not measured, and therefore, individual variability in pharmacokinetics, including potential sex differences in THC absorption, metabolism, and clearance, could not be directly examined. Additionally, further work is needed to compare these machine learning metrics to more demanding, impairment-sensitive cognitive paradigms, including those most relevant to driving impairment specifically.

## Conclusions

This randomized crossover trial of THC vs placebo lays the groundwork for further exploration of fNIRS as a tool for detecting impairment. Future research to validate these findings in larger, more diverse populations, including those using multiple substances, is needed and may also afford classifier improvements through the use of more complex models. Finally, integrating real-time fNIRS data with wearable technologies could enable continuous impairment monitoring. Two promising directions for future research are the development of a multimodal array combining electroencephalography, NIRS, and speckle sensing technologies to simultaneously measure cerebral blood flow, oxygenation, and electrical activity,^33,34^ potentially enhancing sensitivity and accuracy of measurements, and creating a remote sensing system for assessing cerebral blood flow.

## Notes

Supplement 1. Trial Protocol and Statistical Analysis Plan

Supplement 2. eMethods eFigure 1. An Overview of Model Architectures Used eTable 1. Baseline Characteristics eTable 2. Participant Counts of THC Impairment Status and Scan Type eTable 3. Effects of Study Medication on THC Impairment eTable 4. Statistical Comparison of fNIRS Resting-State and n-Back Models Across Classification Metrics eFigure 2. Visual Plots of Impairment Scores, Heart Rate Changes, and Subjective Impairment Ratings per Condition eFigure 3. Within-Individual Performance of the Prediction Model During Each Scan eTable 5. Performance Metrics for Participants Impaired at Only One Post-THC Scan eTable 6. Performance of All Models eFigure 4. Impairment Scores from Resting-State fNIRS Across Demographic and Cannabis-Use Subgroups eFigure 5. Channel-Wise ROC–AUC Scores for Resting-State and N-Back fNIRS Models eReferences

Supplement 3. Data Sharing Statement
