# Supplementary material for: Detection of Δ9-Tetrahydrocannabinol Impairment Using Resting-State Functional Near-Infrared Spectroscopy: A Randomized Clinical Trial
Source: JAMA Netw Open. 2026 Jan 30;9(1):e2556647. doi: 10.1001/jamanetworkopen.2025.56647 (PMC12859723; doi:10.1001/jamanetworkopen.2025.56647)
Supplement: Supplement 1. — Trial Protocol and Statistical Analysis Plan [file jamanetwopen-e2556647-s001.pdf]

PARTNERS HUMAN RESEARCH COMMITTEE

DETAILED PROTOCOL: Using Imaging to Assess Effects of THC on Brain Activity
Protocol #: 2015P001516
Principal Investigator: Jodi Gilman, Ph.D.
Co-Investigator: A. Eden Evins, MD
Version Date: 11/6/2024

I. BACKGROUND AND SIGNIFICANCE

Marijuana has become the most popular illicit drug in the western world (Substance Abuse and Mental Health Services Administration, 2004). Both acute and chronic exposure to cannabis is associated with changes in brain activity in frontal, limbic and also cerebellar regions, with generally increased brain activity following acute exposure and generally decreased activity during abstinence (Quickfall, 2006). Regions showing differences in brain activity involve the extended dopamine reward pathways and the frontocerebellar network.

Most brain imaging studies of acute marijuana exposure have demonstrated global cortical activity increases during administration of smoked cannabis or infused THC. Several techniques have been used to track alterations of the brain associated with marijuana intoxication (Mathew and Wilson, 1992, Levin, 2001 and Mathew et al., 1997). Using positron emission tomography (PET), studies have shown that marijuana intoxication is associated with global and rCBF increases that are most notable over the frontal regions and, in right-handed subjects, in the right hemisphere, (Mathew et al., 1997). Ratings of intoxication and euphoria correlated with the right hemispheric rCBF increase (Mathew et al., 1997). In another PET study, Volkow et al. (1991a) showed that increased cerebellar metabolism while intoxicated correlated with intoxication and plasma THC levels. In another report (Volkow et al.,1991b). FMRI studies have also shown increases in brain regions when individuals are intoxicated. In two pharmacological fMRI studies, Bossong and colleagues investigated the acute effects of THC, administered with a Volcano vaporizer (6 mg), and found that activity during a memory task was significantly increased in a network of memory-related brain regions, particularly when workload increased.

PET and FMRI both have limited ability be used in real-world settings. An alternative technology that has not been used to assess the effects of THC on brain function is functional near-infrared spectroscopy (fNIRS). fNIRS is a neuroimaging technology for mapping the functioning human cortex that exploits the principles of near-infrared spectroscopy (NIRS). An fNIRS headset can be quickly fixed to the forehead and enables measurements of the prefrontal cortex (PFC) within minutes, while guaranteeing high data quality. NIRS is based on the fact that: 1) human tissues are relatively transparent to light in the NIR spectral window (650–1000 nm); 2) NIR light is either absorbed by pigmented compounds (chromophores) or scattered in tissues; 3) NIR light is able to penetrate human tissues (Delpy and Cope, 1997); and 4) the

relatively high attenuation of NIR light in tissue is due to the main chromophore, hemoglobin (the oxygen transport red blood cell protein), located in small vessels of the microcirculation, such as capillary, arteriolar and venular beds. NIRS is weakly sensitive to blood vessels >1 mm because they completely absorb the light. The absorption spectrum of hemoglobin depends on its level of oxygenation. NIRS is a non-invasive, safe technique that utilizes 1) laser diode and/or light emitting diode light sources spanning the optical window between 650 and 1000 nm, and 2) flexible fiber optics to carry the NIR light to (source) and from (detector) tissues. Fiber optics are suitable for any head position and posture. Adequate depth of NIR light penetration (almost one half of the source-detector distance) can be achieved using a source-detector distance around 3 cm. So far, fNIRS has been promoted in a number of fields in which functional magnetic resonance imaging (fMRI) is limited due to the constraints induced by the scanning environment. These devices can be utilized, for example, in infant and children developmental studies, in neurorehabilitation assessment, and in simultaneous brain activation studies on multiple subjects. Finally, the fNIRS field is continuing to grow as more researchers from diverse fields engage the technology. In the current study, we will use fNIRS to assess the effects of THC intoxication on the prefrontal cortex.

Dronabinol (marinol) is an FDA approved, synthetic, orally active cannabinoid that has been shown to be effective for appetite stimulation and safe for chronic use at doses up to 210 mg per day, although after dosing of 210 mg per day for 16 days a withdrawal syndrome was described with abrupt discontinuation. Doses of 2.5-20 mg per day are used for appetite stimulation and antiemetic indications. Doses of 0.5 mg/kg body weight reliably produce intoxication (easy laughing, elation and subjective heightened awareness)

II. SPECIFIC AIMS

Aim 1. Assess the effects of THC intoxication using the medication dronabinol (synthetic THC) on resting state and task-based activation in the prefrontal cortex (PFC), as well as on neurocognitive task performance and correlations between these measurements and clinical signs of intoxication.

Aim 2. Investigate at which doses of dronabinol an effect on neurocognitive task performance or an effect on PFC activity can be observed.

Aim 3. Examine potential interaction following co-administration of THC with oral ethanol exposure in healthy volunteers.

Before enrolling patients in the dronabinol trial, we will enroll 20 participants in Phase 0, in order to pilot the cognitive tasks in order to determine which tasks optimally activate the PFC. In Phase 0, participants will undergo one hour-long fNIRS testing session without taking a dronabinol capsule. Inclusion criteria will be identical to Phase 1, Phase

2 and Phase 3, EXCEPT that participants will not be required to be regular marijuana users.

III. PARTICIPANT SELECTION

Inclusion Criteria

***General* (Phase 2A, 2B 3)**

1. Men and women aged 18-55 years, inclusive; (for Phase 2B: men and women aged 21-55 years, inclusive)
2. Competent and willing to provide written informed consent;
3. Able to communicate in English language.
4. Regular, at least monthly, marijuana use, confirmed by positive urine screen for THC

Additional Inclusion Criteria For Phase 2B:

5. Past consumption of at least two alcoholic beverages in one occasion.
6. Past co-consumption of alcohol and THC at least once in lifetime with no serious adverse effects.
7. Weigh more than 100 lbs.

Exclusion Criteria:

***General* (Phase 2A, 2B 3)**

1. Any unstable, serious medical illness, or cardiovascular disease or events.
2. New or unstable psychiatric symptoms, schizophrenia, or bipolar I disorder,
3. Diabetes, cirrhosis, renal failure, Hepatitis C, HIV,
4. History of syncope without an identified situational stressor, migraines >1x/month, head injury with prolonged unconsciousness (> 24 hours);
5. Allergy to sesame oil (contained in Marinol pills) or Marinol capsules
6. Daily use of benzodiazepines or barbiturates, antihistamines, atropine, scopolamine, or other strong anticholinergic agents;
7. Current pregnancy or lactation, or trying to become pregnant (confirmed by urine pregnancy test)

6\. In the opinion of the investigator, not able to safely participate in this study.

Additional Exclusion Criteria For Phase 2B:

5. Currently seeking treatment, in treatment, or in recovery from an alcohol use disorder.

Tachyphylaxis and tolerance develop to the sympathomimetic effects of dronabinol and other cannabinoids with chronic use, suggesting an indirect effect on sympathetic neurons. Because inclusion criteria require regular cannabis use, and positive THC consistent with cannabis use in the past 3 weeks, we expect participants to have tolerance to the cardiovascular and subjective adverse CNS effects of dronabinol. Nevertheless, we will exclude potential participants with clinically significant cardiovascular disease.

IV. RECRUITMENT METHODS
Participants will be recruited by study staff at the MGH Center for Addiction Medicine through advertising by email, web and bulletin board announcements posted within the local site network community. We will place a Partners HRC approved research study advertisement on relevant websites and in relevant print publications, as well as on the MBTA.

In accordance with NIH guidelines, efforts will be made to attain a mix of final study participants, in terms of sex and racial/ethnic representation that is reflective of the population of the greater metropolitan area where the recruitment is taking place. For example, for the metropolitan Boston area we will attempt to recruit; 63% white, 26% African American and 11% other. Specifically we anticipate that half of our participants will be male and the other half female and that we will have representation from Asian American, African American and Hispanic minority groups in our final cohorts.

V. REMUNERATION
Phase I: Participants will be paid by check at the completion of the study. Participants will be compensated $10 per hour spent at each visit and will earn an additional $30 for completion of the second fNIRS session per study visit. Each study visit will take approximately 7 hours to complete. $100 is the maximum amount a participant can receive per study visit. Participants will be asked to take public transport or be dropped off to the study visit and a member of our study staff will: (1) arrange car service (e.g. Lyft) that has an approved partnership with MGH, and (2) will escort the participant to the cab at the end of the study visit. Alternatively, it would be acceptable for participants to arrange travel home with a family member or friend. If they choose to do this, a study staff member will ensure that someone is there to bring them home prior to departure. An additional $30 will be paid to participants that come in on short notice (in case of any last-minute cancellations).

Phase IIA: Participants will attend a screening visit and 2 study visits, which require approximately 7-hours of testing. The screening visit will take approximately 3 hours to complete, and participants will be compensated $25. The Visit 1 study visit will be reimbursed at $100 and the Visit 2 study visit will be reimbursed at $100. Subjects will be paid at an hourly rate of $10 per hour if they decide to drop out of the study.

Phase IIB: For Phase IIB, participants will attend a screening visit and 4 study visits, which require approximately 7-hours of testing. The screening visit will take approximately 4 hours to complete, and participants will be compensated $25. The 4 study visits will be reimbursed at $100 per visit plus a $50 bonus at the end for study completion. If participants complete all five study visits, they will receive a total of $475 in the form of a check.

Phase III: Participants will be compensated $10 per hour spent at each visit and will earn an additional $30 for completion of the second fNIRS session per study visit. Each study visit will take approximately 7 hours to complete. $100 is the maximum amount a participant can receive per study visit. Participants will attend 3 study sessions, which

require approximately 7-hours of testing. If participants complete all three study visits, they will receive a total of $300 in the form of a check.

VI. PARTICIPANT ENROLLMENT
MGH Center for Addiction Medicine study staff will conduct telephone screening of approximately 2000 potential subjects. A telephone screening will distinguish the majority of likely eligible subjects from those not likely to meet eligibility criteria. This will consist of a brief discussion of the research study, confirming a potential participant's interest in participation and whether he/she meets eligibility criteria and includes asking about cannabis use, current medications, sex, age, pregnancy status, and psychiatric/medical conditions. Screening takes place over the phone in response to a potential participant inquiry (see attached screening telephone script). Those who are not eligible for the experiment based on the phone screen will be informed that they do not qualify for entry into this particular study. The research study consent form will be made available to the participants on their study visit before any study procedures occur. Participants who give permission to receive text messages from the study staff will receive appointment reminders via text through Apptoto three, two, and 1 day(s) before upcoming appointments.

A non-identifying prescreening log will be kept for all individuals screened. The screening log will include a non-identifying subject ID, the date of screening, whether eligibility criteria were met, and enrollment decision or status.

VII. INFORMED CONSENT
Participants who are deemed potentially eligible on the basis of the phone screen will be asked to come in for a study visit. At the time of such visit, informed consent will be obtained by a licensed physician investigator or non-physician investigator with physician back-up prior to administering any study procedures. All participants will be given the opportunity to ask questions to a physician Investigator (Dr. Eden Evins, or another study physician), and this will be recorded in research records. Contact information of key study staff will be provided and participants will be informed that the co-investigators are available to answer any questions or concerns they may have about the study. All eligible participants will be provided with a copy of their signed consent form.

VIII. PARTICIPANT WITHDRAWAL
Participation is voluntary, and participants may stop being in the study at any time or decide not to join the study. If a participant decides not to participate, they will not be penalized in any way, and will not lose any benefits to which they are otherwise entitled.

IX. CONFIDENTIALITY
All phases and aspects of this project will be conducted according to the Declaration of Helsinki and will comply with HIPAA regulations. The sponsors of this research will only have access to data on individuals that is stripped of all unique identifiers according to HIPAA guidelines.

X. STUDY PROCEDURES

Phase 0. *Pilot a number of cognitive tasks using fNIRS, in order to determine which neurocognitive tasks produce reliable activation of the prefrontal cortex (PFC). 20 adults (age 18-55) will be recruited to participate in this phase of the study. These participants are not required to have used marijuana, since they are not receiving dronabinol* capsules.

Phase 1. *Assess the effects of THC intoxication using the medication Marinol (synthetic THC) on resting state and task-based activation in the prefrontal cortex (PFC), as well as on task performance, using a number of computerized neurocognitive tasks.*

<u>Participants</u>: 100 adults (age 18-55) will be recruited to participate in this phase of the study. Approximately half will be occasional marijuana users (use marijuana at least monthly but not more than weekly), and half will be heavy marijuana users (use marijuana more than once per week). Subjects will be asked to refrain from using marijuana within 24 hours of the study. Since THCCOOH, the main secondary metabolite of tetrahydrocannabinol (THC), can be detected in urine several weeks after last use, we will ensure that no participant exhibits overt signs of intoxication (e.g. congestion of the conjunctival blood vessels (red eyes), slowed speech response, giddiness).

<u>Measures</u>: Please see table below for questionnaires:

[redacted]	
Baseline Questionnaires	
Frequency of substance use	WHO ASSIST[1]
Harmful alcohol use	AUDIT[2]
Alcohol consumption urge	AUQ[3]
Estimate of alcohol/marijuana use	Timeline follow-back[4]
Expectancy of marijuana effects	MEEQ,[5]
Marijuana use severity	CUDIT[6]
Marijuana craving	Modified AUQ [3]
State/Trait Anxiety Index	STAI
Hospital Anxiety and Depression Scale	HADS
Insomnia symptoms	PSQI
Cannabis withdrawal	CWS
Consonant Trigrams Test	CTT
Divided Attention Task	DAT
Repeated Assessments During Protocol	
Drug Effects Questionnaire	DEQ
Biphasic Alcohol Effects Scale (modified)	BAES

*All questionnaires will be filled out by participants on study laptops.

Cognitive Tasks during FNIRS testing: We propose to investigate neural activity during various attention, decision-making, language, sensory, affective or emotional processing tasks, as prompted by established task paradigms that have been validated and published under IRB approval from Partner's scientists, or IRBs at other institutions. For the first 6 minutes of each fNIRS scan, participants close their eyes and rest. For the next 6 minutes of each fNIRS scan, participants complete the 0-back and the 2-back condition of the letter N-back working memory (WM) task. During the 0-back condition, participants are instructed to press a response button whenever a letter "X" appeared on the screen. For the 2-back condition, participants are instructed to press the button whenever the presented letter was identical to the letter presented two trials prior. Starting with the 2-back, blocks alternate between the 2-back and 0-back, in which instructions on the screen indicated whether participants should press for the 0-back or 2-back target. Stimuli are generated, and responses were collected using PsychoPy (Psychophysics Software in Python).

Since the fNIRS device meets the criteria for a nonsignificant risk device, we will comply with the abbreviated IDE requirements under §812.2 (b):

I. Labeling - The device will be labeled in accordance with the labeling provisions of the IDE regulations (§812.5) and will bear the statement "CAUTION - Investigational Device. Limited by Federal (or United States) law to investigational use.";

II. IRB Approval – we will obtain and maintain Investigational Review Board (IRB) approval throughout the investigation as a nonsignificant risk device study;

III. Informed Consent – we will obtain and document informed consent from each subject according to 21 CFR 50, Protection of Human Subjects,

IV. Monitoring - All investigations will be properly monitored to protect the human subjects and assure compliance with approved protocols (§812.46).

V. Records and Reports – We will maintain specific records and make certain reports as required by the IDE regulations.

Reports:

A. Unanticipated Adverse Device Effects: we will submit to the sponsor and the reviewing IRB a report of any unanticipated adverse device effect as soon as possible but no later than 5 working days / 7 calendar days after the investigator first learns of the effect.

B. Withdrawal of IRB Approval: we will report to the sponsor a withdrawal of approval of the reviewing IRB within 5 working days.

C. Progress Reports: we will submit progress reports to the sponsor, the monitor, and the reviewing IRB at regular intervals but no less than on a yearly basis.

D. Deviations from the Investigational Plan: we will notify the sponsor and the reviewing IRB of any deviation from the investigational plan to protect the life or physical well-being of a subject in an emergency. The notice must be provided as soon as possible but no later than 5 working days after the emergency occurred. If it is not an emergency, prior approval from the sponsor is required for changes in or deviations from the investigational plan. If the change or deviation may affect the scientific soundness of the investigational plan or the rights, safety or welfare of the subject, the sponsor is required to obtain prior IRB approval and also to obtain FDA approval for a significant risk device investigation by submitting an IDE supplement.

1. Informed Consent: If the device is used without obtaining informed consent, we will report this to the sponsor and to the reviewing IRB within 5 working days after the use occurs.
2. Final Report: we will submit a final report to the sponsor and to the reviewing IRB within 3 months after termination or completion of the investigation.
3. Other Reports: We will provide accurate, complete, and current information about any aspect of the investigation upon request from the reviewing IRB or FDA.

Records: The following records will be maintained in one location and available for FDA inspection under §812.2(b):

- the name and intended use of the device
- the objectives of the investigation
- a brief explanation of why the device is not a significant risk device
- the name and address of each investigator
- the name and address of each IRB
- a statement of the extent to which the good manufacturing practices (21 CFR 820) will be followed in manufacturing the device

Study Drug and Dosing: Participants will be given up to 50mg of MARINOL® (dronabinol) in all phases except Phase IIA and IIB, in which they will be given up to 80mg of dronabinol. Dronabinol is an FDA-approved synthetic form of cannabis that is used to treat loss of appetite that causes weight loss in people with AIDS. Dronabinol contains synthetic THC, the principle psychoactive drug in marijuana. The study physician/nurse and co-investigator, Dr. A. Eden Evins MD, as well as physicians/nurse on study staff at the Center for Addiction Medicine, will be responsible for administering the drug.

Dronabinol will be dosed at up to 80mg. The rationale for this dose is based on several factors. The study will be conducted in regular cannabis users who present at their first study visit with a positive urine screen for THC metabolites. Chronic cannabis use is associated with significant tachyphylaxis and tolerance to pharmacologic effects of THC and other cannabinoids. In healthy males who received 210 mg/day dronabinol for 16 days, tolerance developed to the CNS effects of dronabinol (including the euphoric ‘high’ effect) within 12 days. At doses of 5 mg/day for appetite stimulation in 139 patients, only 18% experienced ay CNS effect (feeling high, dizziness, confusion, somnolence). These CNS effects are the target symptoms we wish to assess in study participants. Because of tolerance, the rate of CNS effects is expected to be lower in our study population than in non-cannabis users who participated in marinol studies for anti-emetic and appetite stimulation indications.

Marinol was evaluated in 454 patients with cancer at doses up to 40 mg/day. At doses greater than 0.175 mg/kg approximately 58% reported feeling ‘high’. The starting recommended antiemetic dose is 15-20mg/day, noting that dosage may be escalated during a chemotherapy cycle or at subsequent cycles based upon initial results.

For appetite stimulation, the recommended dose range is up to 20 mg/day, and at this dose approximately half of patients tested have reported CNS symptoms targeted in this study (feeling high, dizziness, confusion, somnolence).

Thus, we will dose at up to 80mg and will assess for CNS symptoms frequently during the study visit. The exact dronabinol dose will be clinically determined by a study physician. The dose will be designed to minimize adverse psychologic and physiologic effects of dronabinol and will be primarily based on the degree of tolerance expected (based on dose and frequency of cannabis use), self report of degree of intoxication experienced with each use, and any regular or prior negative psychological reactions to cannabis use or binges. This is an inexact determination as potency of cannabis used is almost never known. Other factors that will be considered when determining dose are height, weight, BMI, and baseline blood pressure. For example: Heavy daily cannabis users will generally receive 80mg. Daily cannabis users who use 1/2-1 gram per day and who weigh over approximately 140 pounds will generally receive 50mg, while those who use less than half a gram per day, use less than daily or who weigh less than 100 pounds will receive between 30 and 45 mg based on the physician's best judgment.

Urine Drug Screen: Before study procedures are initiated, we will perform a qualitative urine drug screen (Multi Drug 6 Panel Urine Test; Medimpex United Inc., Bensalem, PA) that will test for marijuana, amphetamines, methamphetamines, cocaine, and opiates. These rapid tests are simple, can be done at home, and are CLIA-waived. The test will be administered by a trained research coordinator.

Urine Pregnancy: Before study procedures are initiated, we will perform a qualitative urine pregnancy test for females. The test will be administered by a trained research coordinator.

Overview of Procedures for Each Phase:

Phase 0: Participants will come to the MGH Martinos Center the day of their study visit. They will meet with a study doctor and sign the consent form. Those who are eligible after the telephone screen will then undergo one fNIRS testing session. The testing session will take approximately one hour to complete. The testing procedure only consists of the n-back task (see "Cognitive Tasks during FNIRS testing" for description of the task). There is no drug administration in Phase 0.

Phase 1: Participants will come to the MGH Martinos Center the morning of their study day. They will meet with a study doctor, sign the consent form, and complete screening for eligibility. Screening consists of a substance use history interview (timeline follow back), medical history interview, and a urine drug and pregnancy test. Those who are eligible will then complete baseline questionnaires, including the DEQ and BAES. Participants will be trained to become familiarized with fNIRS tasks and undergo the first fNIRS testing session. They will then be given a dose of dronabinol. Every 20 minutes,

they will complete the DEQ and BAES. fNIRS testing will be repeated at 100 min (thus the second fNIRS session will occur over 60 minutes comprising the timespan of 90-150 minutes after ingesting dronabinol, which is the median peak of pharmacokinetic effects). Participants will then have a one-hour break and undergo an optional third fNIRS testing session at 210-270 min (3.5-4.5 hrs after ingesting dronabinol, when expected effects have dissipated and the person is no longer clinically intoxicated) if time allows. Although not expected, if repeat fNIRS results at 210 min are consistent with continued intoxication, in order to obtain measurements following clinical intoxication, we may repeat the fNIRS testing at 330 minutes, 1.5 hours outside the peak window of intoxication).

The fNIRS device will only be operated by individuals who have received laser use training: Dr. Jodi Gilman, Rachel Plummer, Olivia Downer, Audrey Steely, Rachel Marcus, and Nathaniel Phillips.
See below for schematic:

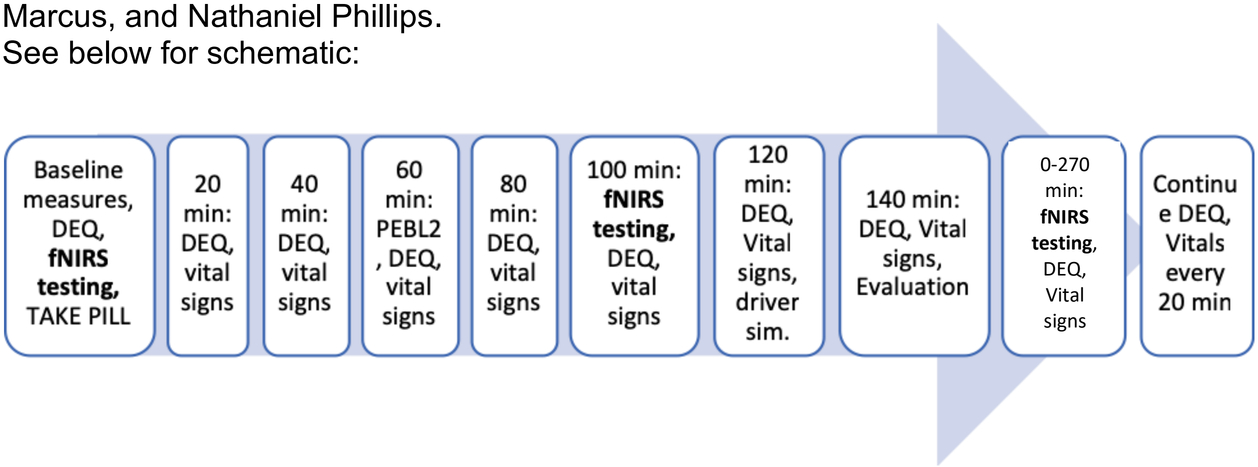

In between testing sessions, participants will be allowed to read, use a laptop, or watch movies. They will be provided with snacks and drinks.

Phase 0 is expected to take approximately 2 months to complete. Phase 1 is expected to take approximately 1 year to complete. Phase 2 will begin after Phase 1 data collection is completed, and is expected to take approximately 1 year. Phase 3 data collection will start after completion of Phase 2, and is also expected to take approximately 1 year.

Phase 2A. *Investigate the effect of THC on fNIRS brain signature and its association with self-reported intoxication, laboratory measures of impairment, and the gold-standard behavioral field test of driving impairment used by law enforcement, the primary classifier.*

Participants will come to 101 Merrimac Street for 3 study sessions. At the first visit, participants will undergo screening procedures to determine if they're eligible for the study. This will include a medical history interview, substance use history interview (timeline follow back), as well as a variety of questionnaires (to be completed by participants on study laptop; AUDIT, CUDIT, STAI, HADS, URGE, MEEQ, Who Assist, PSQI, CWS). Participant vital signs, height, and weight will be measured, and

participants will also provide a urine sample which will be tested for drugs and pregnancy (if applicable) to ensure participants are positive for THC. Participants are trained on the driving simulator computer tasks. If participants are determined to be eligible, they will be scheduled for the following two visits. In a double-blind, placebo-controlled, random order cross-over study of single dose THC (dronabinol) at physician determined doses of 10-80mg, participants will receive THC capsules or identical placebo on two separate study visits, randomized for order. The MGH research pharmacy will generate a blinded randomization code for order of dosing and will dispense blinded drug in the dose ordered and identical placebo for use on separate study days. In order for a complete washout of effects, and to minimize the effect of repeat testing for the neuropsychology tasks, study visits must be at least 1 week apart. On each study day, participants will have a light standardized breakfast after overnight fast and sleep. They will be asked not to deviate from normal behaviors (e.g. if they drink caffeinated beverages or smoke cigarettes in the mornings, they will be asked to do so on the study day so that no symptoms of caffeine or nicotine withdrawal will appear during the scanning period. On each study day, participants will undergo baseline fNIRS testing (see above "Cognitive Tasks during FNIRS testing"), substance use history interview (timeline follow back), two sets of baseline vital signs, a driving simulator computer task refresher, and several questionnaires (HADS, URGE, PSQI, CWS). Dronabinol or placebo will be administered once morning procedures are complete, after which point heart rate, blood pressure, and responses to DEQ and BAES questionnaires will be monitored at 20 minute intervals. The second fNIRS session will be conducted at approximately 90 minutes post dose, followed by driving simulator computer tasks. A field test of sobriety will be conducted immediately following the second fNIRS assessment. The third fNIRS session will be conducted at approximately 220 minutes post dose, prior to participant discharge. See Below for schematic:

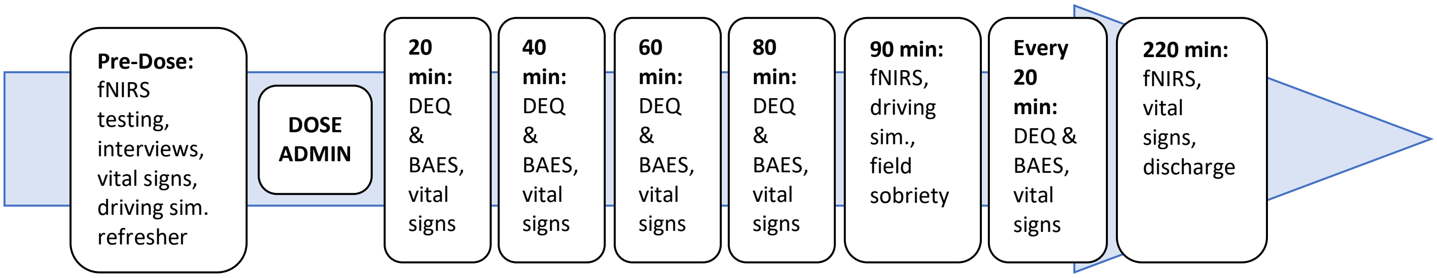

Subjects who participated in Phase 1 will be eligible for Phase 2, but enrollment in Phase 1 is not a requirement for Phase 2. Therefore, new subjects who have not completed Phase 1 will also be eligible for Phase 2.

Driving Simulation: During this phase, we will use a computer-programmed driving simulator to assess motor coordination of the participants. The simulation will programmed in STISIM on the computer and will be administered by a trained research coordinator. Subjects will be trained on this task during the screening task during the screening visit and given a refresher training on the task before testing. The task is performed on a laptop with the use of the keyboard, a joystick, and a foot pedal.

Participants perform tasks where they must center an object on the screen using the joystick, in addition to identifying numbers with the tap of the foot pedal.

Participants: 200 healthy, weekly or greater MJ users, aged 18-55, will be enrolled. Participants must test positive for cannabis and have no serious unstable medical illness to participate in the Phase II (See Participant Selection Section).

***Phase 2B**. Examine potential interaction following co-administration of THC with oral ethanol exposure in healthy volunteers.*
[Design adapted from recently published alcohol interaction studies (Modi et al., 2007; Trevisan et al., 2008).]

Phase 2B is a randomized, double-blind, 4-treatment, 4-period, crossover study with THC or placebo administration and ethanol or placebo administration. On each of the 4 study days, participants will be admitted to the Translational and Clinical Research Center (TCRC) at Massachusetts General Hospital or the Center for Addiction Medicine (CAM). All visits in the TCRC and CAM will be monitored by licensed nursing staff. We will provide a breakfast of approximately 400 calories (e.g. juice, cereal, bagel), before study procedures begin.

Participants will be assigned randomly to 1 of 4 sequences, and receive each of the following treatments:

- Placebo THC + Ethanol
- THC + Placebo Ethanol
- THC + Ethanol
- Placebo THC + Placebo Ethanol

We will ensure a washout of at least 1 week between periods.

The process of THC dosing and administration will similar to those as described in Phase 2A. Participants will come to 101 Merrimac Street for 5 study sessions. At the first visit, participants will undergo screening procedures to determine if they're eligible for the study. This will include a medical history interview, substance use history interview (timeline follow back), as well as a variety of questionnaires (to be completed by participants on study laptop; AUDIT, CUDIT, STAI, HADS, URGE, MEEQ, Who Assist). Participant vital signs, height, and weight will be measured, and participants will also provide a urine sample which will be tested for drugs and pregnancy (if applicable) to ensure participants are positive for THC. Participants are trained on the driving simulator computer tasks. If participants are determined to be eligible, they will be scheduled for the following two visits. A study physician will use the information from the medical history to determine the dronabinol dose. In addition to this, the physician/nurse will also collect a carbon monoxide (CO) measurement to biochemically establish smoking status. Dronabinol administration at visits 1-4 in the TCRC or CAM will be the same as described in phase 2A. On each study day, participants will undergo baseline fNIRS testing (see above "Cognitive Tasks during FNIRS testing"), substance use history interview (timeline follow back), baseline vital signs and weight measurement, a

driving simulator computer task refresher, and several questionnaires (PSQI, CWS). Dronabinol or placebo will be administered once morning procedures are complete, after which point heart rate, blood pressure, and responses to DEQ and BAES questionnaires will be monitored at 20 minute intervals. ETOH or placebo will be administered 60 minutes after dronabinol or placebo. The second fNIRS session will be conducted at approximately 100 minutes post dose, followed by driving simulator computer tasks. A field test of sobriety will be conducted following the second fNIRS assessment. The third fNIRS session will be conducted at approximately 280 minutes post dose, prior to participant discharge. See below for schematic:

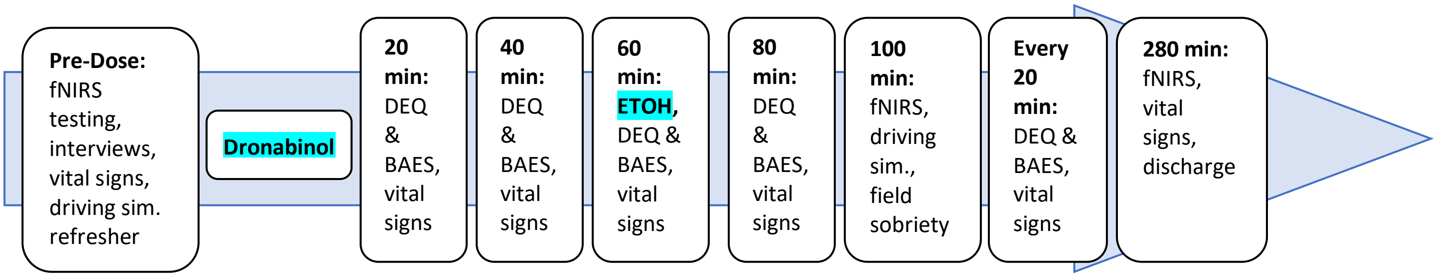

Ethanol dose and administration: We will obtain a breath alcohol concentration (BrAC) of approximately 0.05 BrAC (equal to 1-2 standard drinks). In order to obtain this precise BrAC, we will use established methods of dosing based on previously published procedures (e.g. King 2005, Alcohol Clin Exp Res, Vol 29, No 4, 2005: pp 547–552).

The required volume of 95% ethanol will be calculated from the nomogram published by Watson (1989), and diluted to a final concentration of 20% by volume with diet soda. The nomogram is based on total body water, estimated for each subject using standard equations based on gender, age, height, and weight (Watson et al., 1980). Doses will be prepared at the TCRC by a licensed nutritionist or at CAM by unblinded study staff. He/she will determine the dose according to our spreadsheet for dosing (attached).

In an attempt to standardize the drinking time to 8 minutes, the total dose will be split into 4 equal aliquots and placed in cups with lids. The subject will be given a cup every 2 minutes with instructions to sip the drink using a straw.

- Placebo Ethanol drink will be made as an equal volume of diet soda placed in identical cups with lids and straws. 0.25 ml of 95% ethanol should be floated on the top of the soda in each cup in an attempt to blind the participant.

The alcohol administration procedure will take place approximately 45 min before the start of the second fNIRS session. Before being administered alcohol, study staff will measure breath alcohol levels. Additionally, a study physician/nurse will assess subjects at each study visit prior to any study drug administration for signs of intoxication. Subjects will be terminated from the study if BrAC > 0 or if they exhibit clinical signs of intoxication at time of arrival for study visit.

During this phase, breathalyzer measurements will be taken approximately every 20 minutes after the alcohol administration procedure, until BrACs have returned to <0.01. Participants will be required to stay in the laboratory until BrACs have returned to <0.01. A member of our study staff will then (1) arrange cab service that has an approved partnership with MGH, and (2) escort the participant to the cab at the end of the study visit. Alternatively, it would be acceptable for participants to arrange travel home with a family member or friend. If they choose to do this, a study staff member will ensure that someone is there to bring them home prior to departure.

The dronabinol capsules will be taken approximately 60 minutes before the alcohol administration procedure will begin, so that peak effects of each drug will be concurrent.

Driving Simulation: During this phase, we will use a computer-programmed driving simulator to assess motor coordination of the participants. The simulation will programmed in STISIM on the computer and will be administered by a trained research coordinator. Subjects will be trained on this task during the screening task during the screening visit and given a refresher training on the task before testing. The task is performed on a laptop with the use of the keyboard, a joystick, and a foot pedal. Participants perform tasks where they must center an object on the screen using the joystick, in addition to identifying numbers with the tap of the foot pedal.

Blood Draw: During this phase, we will collect blood samples to track THC levels throughout the study. All blood samples will be taken by a catheter inserted in a forearm vein by a licensed nurse. One mL of blood will be taken pre-dose and approximately every 20-40 minutes post-dose until 300 minutes post-dose: 0, 40, 80, 100, 130, 160, 190, 210, 260, 300, for a total of 10 mL. Collected blood samples will be processed by an outside company, Immunalysis Corporation, to test for plasma concentrations of delta-9 THC.

Participants: 200 healthy, weekly or greater MJ users, aged 21-55, will be enrolled. Participants must test positive for cannabis and have no serious unstable medical illness to participate in Phase 2B (See Participant Selection Section).

Subjects who participated in Phase 1 or 2A will be eligible for Phase 2B, but enrollment in Phase 1 or 2A is not a requirement for Phase 2B. Therefore, new subjects who have not completed Phase 1 or 2A will also be eligible for Phase 2B so long as they are over the age of 21. Subjects who enroll in more than one phase of the study will be (1) re-consented for each phase and (2) counted again toward enrollment targets (i.e., counted toward the enrollment target listed for each study phase).

Phase 3. *Investigate at which doses of dronabinol an effect on neurocognitive task performance or an effect on PFC activity can be observed.*

Procedures will be identical to those of Phase 1 except that subjects will be scheduled for 3 study sessions. In each study session, they will be given a low, medium, or high

dose of dronabinol (20 mg, 35 mg, 50 mg). In this single-blinded phase, the subject will not know which dose he/she is receiving. The protocol for the study visit will be identical. As there are three levels of drug exposures, there are 6 possible orders in which subjects can receive the three doses (e.g. high, medium, low; medium, low, high; etc). Subjects will be randomly assigned to one of the 6 possible orders according to a randomization schedule generated by the study staff using a random number generator and computer program. In order for a complete washout of effects, and to minimize the effect of repeat testing for the neuropsychology tasks, study visits must be at least 1 week apart. See below for schematic:

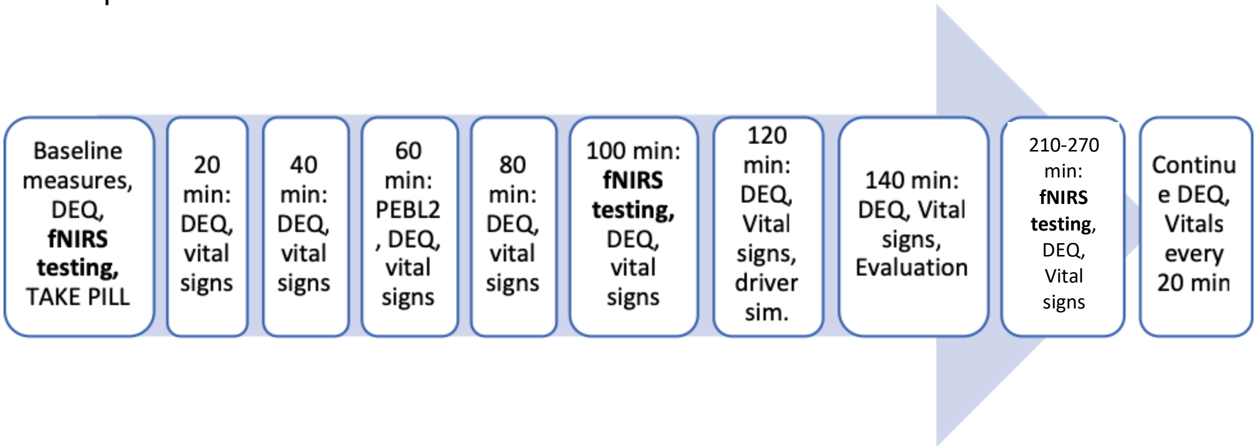

Subjects who participated in Phase 1 or 2 will be eligible for Phase 3, but enrollment in Phase 1 or 2 is not a requirement for Phase 3. Therefore, new subjects who have not completed Phase 1 or 2 will also be eligible for Phase 3.

Driving Simulation: During this phase, we will use a computer-programmed driving simulator to assess motor coordination of the participants. The simulation will programmed in STISIM on the computer and will be administered by a trained research coordinator. Subjects will be trained on this task during the screening task during the screening visit and given a refresher training on the task before testing. The task is performed on a laptop with the use of the keyboard, a joystick, and a foot pedal. Participants perform tasks where they must center an object on the screen using the joystick, in addition to identifying numbers with the tap of the foot pedal.

Participants: 60 healthy, weekly or greater MJ users, aged 18-55, will be enrolled. Participants must test positive for cannabis, and negative for other addictive drugs and have no serious unstable medical illness to participate in the Phase III (See Participant Selection Section).

Biostatistical Analysis

Data variables collected for study

Standardized questionnaires, assessing drug use, personality characteristics, and anxiety will be collected, as well as task data and neural signaling data from the fNIRS testing sessions.

All data will be collected and managed using REDCap electronic data capture tools (Harris 2009) hosted with Partners HealthCare.

Vanderbilt University, with collaboration from a consortium of institutional partners, has developed a software toolset and workflow methodology for electronic collection and management of research and clinical trial data. REDCap (Research Electronic Data Capture) data collection projects rely on a thorough study-specific data dictionary defined in an iterative self-documenting process by all members of the research team with planning assistance from Partners HealthCare Research Computing, Enterprise Research Infrastructure & Services (ERIS) group. The REDCap Survey is a powerful tool for building and managing online surveys. The research team can create and design surveys in a web browser and engage potential respondents using a variety of notification methods. Both REDCap and REDCap Survey systems provide secure, HIPAA compliant, web-based applications that are flexible enough to be used for a variety of types of research, provide an intuitive interface for users to enter data and have real time validation rules (with automated data type and range checks) at the time of entry.

fNIRS Data Analysis

The instrument will acquire raw data for each detector at ~40 kHz. The individual source signals then will be obtained with a digital bandpass filter – for example, a discrete Fourier transform or an infinite-impulse-response filter. The individual source signals will be separated off-line with an infinite-impulse-response filter with a 50 Hz band pass frequency. Next, the time series pairs from each detector will be converted from wavelength (absorption) to relative concentrations (of oxy-/deoxy-hemoglobin). This will be done with the modified Beer-Lambert Law and the absorption coefficients of oxy- and deoxy-hemoglobin (HbO and Hb, respectively) [34]. (The near-IR wavelengths are chosen because oxy-/deoxy-Hb are the predominant absorbers in the tissue at these wavelengths.) The result will be an oxy-Hb and deoxy-Hb timeseries associated with each source-detector pair.

For each stimulation run, each smoothed oxy-/deoxy-hemoglobin time series will be examined for periodic amplitude fluctuations. Expected fluctuations include heart rate (~1Hz), breathing and/or “v-waves” (~0.1Hz), as well as any fluctuations induced by the experimental paradigm. If the former sources of noise are substantial, an attempt will be made to filter out these fluctuations (in Fourier space). Any analysis of filtered data will be compared to an analysis of the corresponding non-filtered data. The oxy-hemoglobin portion of the experimentally induced changes are expected to be 4-9 sec out of phase with the paradigm onsets and offsets (as seen in fMRI work as well as in prior optical recordings).

Study endpoints

As this is not a clinical trial, we will collect data from up to 20 participants in Phase 0, up to 100 participants in Phase I, up to 200 in Phase II and up to 60 in Phase III in order to investigate whether we can determine the brain signals involved in marijuana intoxication. Given that the planned studies are pilot in nature, and that we will be using

a recently developed instrument, the relevant effect sizes are unknown and therefore power analysis cannot be adequately performed. However, some estimates can be made. The biological basis for the signals measured by the optical technique is the same as the basis for fMRI and PET scanning. In a similar study using PET (*R.J. Mathew et al. / Psychiatry Research: Neuroimaging 116 (2002) 173–185)*, indices of rCBF, intoxication and physiology were measured at baseline and 30, 60, 90 and 120 min. after an intravenous infusion of 0.15 or 0.25 mg of THC, or placebo. Significant increases were seen in global perfusion and in the frontal, insular and anterior cingulate regions. This study had a sample size of 47 subjects in each group. Therefore, we will collect data in 100 participants, as this is a conservative estimate of the power we will need to detect an effect.

<u>Statistical methods</u>
Statistical analysis of data will be performed using SPSS, STATA, and fNIRS analysis software.

Data Sharing Plan
De-identified subject-level data will be shared with Dr. Nisan Ozana at Bar Ilan University to investigate the brain's response to THC and alcohol. Dr. Ozana has previously collaborated with Dr. Gilman (PI) on data analysis in his role as a Postdoctoral Researcher and Visiting Scholar at MGH/HMS. Data elements to be shared include fNIRS data and clinical rating assessments and measures, such as heart rate, pulse, and subjective drug effects. The data will be coded and the external collaborator will not have access to the coded key. Data from approximately 50 subjects will be sent. Data will be sent electronically using an MGB-approved Data Sharing Collaboration Tool (MGB Dropbox for Business/Secure File Transfer). A data use agreement will be executed with the receiving institution.

De-identified subject level data will be shared with collaborators at OBELAB, Inc. and Korean Advanced Institute of Science and Technology (KAIST) to analyze the brain's response to THC. Collaborators include Drs. Jongkwan Choi, Hanseok Yun, and Jaemyoung Kim. Data elements to be shared include the fNIRS neuroimaging data and clinical rating assessments and measures, such as heart rate, pulse, and subjective drug effects. The data will be coded, and the external collaborators will not have access to the coded key. Data from approximately 100 subjects will be sent. Data will be sent electronically using an MGB-approved Data Sharing Collaboration Tool (MGB Dropbox for Business/Secure File Transfer). A data use agreement will be executed with the recipient before data is shared.

Risks and Discomforts

<u>Potential adverse events due to dronabinol:</u>

<u>Risks</u>: Dronabinol-induced sympathomimetic activity may result in tachycardia and/or conjunctival injection. Its effects on blood pressure are inconsistent, but occasional

subjects have experienced orthostatic hypotension and/or syncope upon abrupt standing. Dronabinol also demonstrates reversible effects on appetite, mood, cognition, memory, and perception. These phenomena appear to be dose-related, increasing in frequency with higher dosages, and subject to inter-patient variability.

After oral administration, dronabinol has an onset of action of approximately 0.5 to 1 hours and peak effect at 2 to 4 hours. Duration of action for psychoactive effects is 4 to 6 hours.

Tachyphylaxis and tolerance develop to some of the pharmacologic effects of dronabinol and other cannabinoids with chronic use, suggesting an indirect effect on sympathetic neurons. In a study of the pharmacodynamics of chronic dronabinol exposure, healthy male volunteers (N = 12) received 210 mg/day dronabinol, administered orally in divided doses, for 16 days. An initial tachycardia induced by dronabinol was replaced successively by normal sinus rhythm and then bradycardia. A decrease in supine blood pressure, made worse by standing, was also observed initially. These volunteers developed tolerance to the cardiovascular and subjective adverse CNS effects of dronabinol within 12 days of treatment initiation.

In the clinical trials, the majority of patients were treated with 5 mg/day MARINOL Capsules, although the dosages ranged from 2.5 to 20 mg/day.

Minimization: If there are any concerns about a subject in need of clinical attention, the PI will be made aware of the issue immediately and will consult with appropriate medical personnel (co-investigator: A. Eden Evins, MD, or another study physician) to determine appropriate steps. The PI and medically trained co-investigators will assess the needs of the subject and offer the subject either prompt treatment or medical referral, whichever is appropriate for the situation. There is a licensed physician/registered nurse on site 40 hours per week, and other MGH resources can be used as necessary.

Potential adverse events due to ethanol:
Risks: At low to moderate doses, ethanol can produce behavioral intoxication and physiological changes (feeling intoxicated, high, euphoric, dizzy, giddy, tired and lightheaded; increased heart rate, slurred speech, and slowed reaction time). These effects will not be novel to our subjects who will have experience drinking. The amount of alcohol administered is similar to that experienced by casual users of this drug and is fairly low for moderate to heavy users.
Minimization: Participants will not be required to perform any physical tasks while intoxicated. Drinking alcohol under controlled laboratory conditions is unlikely to change current use patterns or cause addiction. If there are any concerns about a subject in need of clinical attention, the PI will be made aware of the issue immediately and will consult with appropriate medical personnel (co-investigator: A. Eden Evins, MD, or another study physician) to determine appropriate steps. These investigators will handle any adverse reaction (physical or psychological) to the drug administration.

Psychosocial risks
Risks: There is no social risk associated with participation in this study. There are few legal risks to the participants associated with these paradigms. Cannabis use is decriminalized in Massachusetts, thus there is no legal risk to self-report of regular cannabis use. However, a possible risk is that subjects may come up positive on a workplace drug screen if they take part in this study.
Minimization: We will provide subjects with documentation that describes the study procedures, and states that participation in this study will affect any drug screen results.

Confidentiality and Loss of Privacy
Risks: The risk of loss of privacy is judged to be minimal.
Minimization: Confidentiality will be maintained by numerically coding all data and by keeping all data in locked file drawers and deidentified data in password protected databases. Subject information will be accessible only to research staff. Information about study participants will not leave our institution in any form that would identify individual subjects.

fNIRS Risks
Risks: fNIRS is a minimal risk procedure. fNIRS assess the brain's hemodynamic response by employing optical properties of light. Unlike fMRI, fNIRS only collects data ~3 cm into the cortex. fNIRS employs non-ionizing near-infrared light, so no cancerogeneous or genetic effects are possible. The light intensity is kept well below safety limits to avoid the risk of thermal damages. No cumulative harmful effects of such light exposure to the forehead are known, and fNIRS measurements are commonly applied repeatedly or in a continuous manner for monitoring purposes.

Minimization: Subjects who may become uncomfortable during the fNIRS procedure will be able to converse with the experimenter and will be able to communicate any desire or need to stop the procedure. All efforts will be made to make the subjects as comfortable as possible. A member of the study staff will explain the procedure thoroughly to the subject, allowing for maximal understanding and comfort. The testing can be stopped at any time at the subjects' request. A qualified physician/nurse will be on site or reachable by pager during all study visits in the unlikely event that an adverse event occurs.

The likelihood for the need for medical or professional intervention for a participant in this study is reduced by the protocol enrollment criteria that specify that we will enroll only medically stable, healthy adults to the study. Subjects will be asked to report adverse events at any time by calling study staff and will be asked to report adverse events at each study visit.

Risks of blood draws
Risks: The discomfort associated with removing (by catheter from a vein) is a slight pinch or pin prick when the sterile needle enters the skin. The risks include mild discomfort and/or a black and blue mark at the site of puncture. Less common risks include a small blood clot, infection or bleeding at the puncture site, and on rare

occasions fainting during the procedure. There will be only a small amount of blood drawn (about 1 mL) each time.

Minimization: Blood draws will be taken by trained nursing staff at the Translational and Clinical Research Center or Center for Addiction Medicine at Massachusetts General Hospital. The total amount of blood drawn will not exceed 5 ccs per draw or 45 ccs per study day. If there are any concerns about a subject in need of clinical attention, the PI will be made aware of the issue immediately and will consult with appropriate medical personnel to determine appropriate steps.

Potential Benefits

There is no direct benefit to participating in this research. Some participants may find that talking about marijuana use increases their awareness of any issues related to drug use. Any participant who asks about treatment will be provided information regarding local drug treatment programs.

Information developed from this study may help researchers in the future. Specifically it is expected that this study will provide valuable information to the field of actute marijuana effects on the brain.

MONITORING AND QUALITY ASSURANCE

All participants will have contact information for the principal investigator and the study coordinator if they have questions at any time. Dr. Gilman is responsible for the overall management of the study and will maintain regular communication with all of the study staff. The PI will meet weekly with all study investigators to review the details of data acquisition and analysis as well as any minor problems. In the event of any minor or significant adverse event the PI will be contacted immediately. The PI is responsible for the generation of summary reports to the Partners HRC and to NIH, documenting this process and outcome.

Serious adverse events are not expected as a result of the study procedures. Should one occur, it will be reported by telephone or email by the principal investigator to the Partners IRB according to current PHRC Adverse Event Reporting Policy (version dated: March 13, 2007). All adverse events (if not serious) will be reported in writing to the Partner’s Human Research Committee. All information regarding experimental subjects will be kept in the offices of the Principal Investigator. All data will be identified by a unique code number.

Using Imaging to Assess Effects of THC on Brain Activity (fNIRS)

Statistical Analysis Plan

The fNIRS Team

Version 1

First version: 4/29/2022

Latest version: 4/29/2022

ClinicalTrials.gov ID: NCT03655717

Unique protocol ID: 2015P001516

U.S. NIH Grant/Contract Award Number: R42DA043977

Table of contents

Table of contents 2

Glossary 3

Study Design 4

Participants 4

Analytic Approach 5

Primary Outcomes 5

Statistical Model 5

Covariates 6

Missing Data 6

Sample Size Determination 7

Software 8

References 9

Glossary

Abbreviation	Label
ETOH	Alcohol
FDA	Federal Drug Administration
fNIRS	Functional near-infrared spectroscopy
HbO	Oxygenated hemoglobin
PFC	Pre-frontal cortex
THC	Tetrahydrocannabinol

Study Design

This study will assess the effects of THC intoxication using dronabinol (synthetic THC) on the HbO signal during resting state and task-based activation in the prefrontal cortex (PFC), resting state connectivity, neurocognitive task performance, clinical signs of intoxication, and correlations between these measurements. Participants will be given up to 80 mg of dronabinol, an FDA-approved synthetic form of THC that is used to treat loss of appetite that causes weight loss in people with AIDS. THC is the principle psychoactive drug in marijuana. The study will be conducted in regular cannabis users who present at the screening visit with a positive urine screen for THC metabolites. Data will be collected over two phases:

- Phase 2A is a randomized, double-blind, placebo-controlled, study of effect of dronabinol vs placebo. This phase will investigate the effect of THC on fNIRS brain signature and its association with self-reported intoxication, laboratory measures of impairment, and the gold-standard behavioral field test of driving impairment used by law enforcement.
- Phase 2B is a randomized, double-blind, placebo-controlled, 2 by 2 crossover study of effect of dronabinol, ethanol, and combined dronabinol and ethanol on brain activation and connectivity as measured by fNIRS. Phase 2B will examine potential interaction between THC and alcohol following co-administration of THC with oral ethanol exposure in healthy volunteers.

Participants

Participants will be 150 adults who use marijuana at least monthly (aged 18-55) will be recruited to participate in this study.

Analytic Approach

Primary Outcomes

The primary outcomes for the study will be…

1. The mean HbO level averaged over the duration of the N-back task scan (approximately 2938 samples taken over 376 seconds in 128 millisecond intervals).
2. The mean HbO level averaged over the duration of the resting state scan (2814 samples taken over 360 seconds in 128 millisecond intervals).

Mean HbO levels for both outcomes will be determined over (a) four conditions administered over separate visits about a week apart (where participants could be dosed with placebo, THC only, ETOH only, or both THC and ETOH), and (b) two scans per condition: A scan prior to and approximately 100 minutes after being dosed. Higher HbO levels indicate a greater degree of neural activity in the PFC regions of the brain.

Statistical Model

We will analyze both primary outcomes using a multi-level linear regression model with subject-varying intercepts estimated via maximum likelihood. The key exposures are whether a person was dosed with placebo, THC, ETOH, or the combination of THC and ETOH. Table 1 lists the numeric coding used to test our planned contrasts. All terms will be coded as 0 for pre-dose scans, as no combination of drugs will have been administered.

Table 1: Design matrix for testing key exposures

Scan	Dose	Main effects<br>THC	 ETOH	Interaction<br>THC x ETOH
Post-dose	Placebo	0	0	0
	THC	1	0	0
	ETOH	0	1	0
	THC + ETOH	1	1	1

Statistical significance for each contrast will be determined via a non-parametric bootstrap procedure for robustness. First, each subject's set of completed condition (placebo, THC, ETOH, THC + ETOH) will be randomly shuffled across their set of visits. Second, the shuffled data will be refit with the multi-level model, and coefficient estimates will be obtained for the key exposures. Third, the prior two steps will be repeated 10,000 times, providing a null distribution to compare the observed coefficient estimates against. The p-values for the key exposures will be estimated as $P(|o| < |s_i|)$, where o is the observed coefficient, and s_i is the ith coefficient from the shuffled data. For each outcome there will be 3 p-values (the main effects of THC and ETOH and their interaction). The total set of 6 p-values will be adjusted for multiple comparisons using the Benjamini-Hochberg method (Benjamini & Hochberg, 1995). An adjusted p-value < 0.05 will indicate a statistically significant effect of either THC, ETOH, or their interaction on neural activity in the PFC.

Covariates

We will include two sets of covariates in our model. First, we will include a dummy-coded contrast comparing pre-dose scans (coded as 0) to post-dose scans (coded as 1). Second, we will include covariates adjusting for visit order. The referent will be the first visit (coded as -1), and we will include three terms for the second, third, and fourth visit (coded as 1 for the given visit and 0 otherwise). All terms will then be converted to z-scores.

Missing Data

We will only include data from complete visits with both pre-dose and post-dose scans. Participants with no complete pairs of pre and post-dose scans for any conditions will be excluded from the analyses. Missingness will be naturally handled by the multi-level modeling approach, as the higher order terms governing the random effects provide shrinkage and information even in the presence of missing cases (Baayen, Davidson, & Bates, 2008).

Sample Size Determination

Sample size was pre-specified in our grant application. Since no prior studies have attempted to predict impairment using fNIRS measurements, we did not have an effect estimate with which to determine sample size. In order to maximize the chance that standard methods could detect an effect of intoxication on PFC activation patterns with the N-Back task, we collected as many scans as possible with the R42 grant funds allotted.

Software

All analyses will be done using the statistical software R (version 4.1.1; R Core Team, 2021) and integrated development environment RStudio (version 2020.9.0.351; RStudio Team, 2021). Data will be prepared using the R packages ‘dplyr’ (version 1.0.7; Wickham, François, Henry, & Müller, 2021. Models will be fit using the R package ‘lme4 (version 1.1-27.1; Bates, Maechler, Bolker, & Walker, 2015).

References

Baayen, R. H., Davidson, D. J., & Bates, D. M. (2008). Mixed-effects modeling with crossed random effects for subjects and terms. *Journal of Memory and Language*, *59* (4), 390-412. https://doi.org/10.1016/j.jml.2007.12.005

Bates, D., Maechler, M., Bolker, B., & Walker, S. (2015). Fitting linear mixed-effects models using lme4. *Journal of Statistical Software*, *67* (1), 1-48. https://doi.org/10.18637/jss.v067.i01.

Benjamini, Y., & Hochberg, Y. (1995). Controlling the false discovery rate: a practical and powerful approach to multiple testing. *Journal of the Royal Statistical Society: Series B (Methodological)*, *57* (1), 289-300. https://doi.org/10.1111/j.2517-6161.1995.tb02031.x.

R Core Team (2021). R: A language and environment for statistical computing [Computer software]. https://www.R-project.org.

RStudio Team. (2021). *RStudio: Integrated development environment for R* (version 2020.9.0.351) [Computer software]. RStudio, PBC. http://www.rstudio.com/

Wickham, H., François, R., Henry, L. & Müller, K. (2021). *dplyr: A grammar of data manipulation* (version 1.0.7). [Computer software] Retrieved from https://cran.r-project.org/web/packages/dplyr/index.html
