# Supplementary material for: Detection of Δ9-Tetrahydrocannabinol Impairment Using Resting-State Functional Near-Infrared Spectroscopy: A Randomized Clinical Trial
Source: JAMA Netw Open. 2026 Jan 30;9(1):e2556647. doi: 10.1001/jamanetworkopen.2025.56647 (PMC12859723; doi:10.1001/jamanetworkopen.2025.56647)
Supplement: Supplement 2. — eMethods eFigure 1. An Overview of Model Architectures Used eTable 1. Baseline Characteristics eTable 2. Participant Counts of THC Impairment Status and Scan Type eTable 3. Effects of Study Medication on THC Impairment eTable 4. Statistical Comparison of fNIRS Resting-State and n-Back Models Across Classification Metrics eFigure 2. Visual Plots of Impairment Scores, Heart Rate Changes, and Subjective Impairment Ratings per Condition eFigure 3. Within-Individual Performance of the Prediction Model During Each Scan eTable 5. Performance Metrics for Participants Impaired at Only One Post-THC Scan eTable 6. Performance of All Models eFigure 4. Impairment Scores from Resting-State fNIRS Across Demographic and Cannabis-Use Subgroups eFigure 5. Channel-Wise ROC–AUC Scores for Resting-State and N-Back fNIRS Models eReferences [file jamanetwopen-e2556647-s002.pdf]

Supplemental Online Content

Berchansky M, Evans AE, Evohr B, et al. Detection of Δ9-tetrahydrocannabinol impairment using resting-state functional near-infrared spectroscopy: a randomized clinical trial. *JAMA Netw. Open*. 2026;9(1):e2556647. doi:10.1001/jamanetworkopen.2025.56647

eMethods.
eFigure 1. An Overview of Model Architectures Used
eTable 1. Baseline Characteristics
eTable 2. Baseline Characteristics
eTable 2. Participant Counts of THC Impairment Status and Scan Type
eTable 3. Effects of Study Medication on THC Impairment
eTable 4. Statistical Comparison of fNIRS Resting-State and n-Back Models Across Classification Metrics
eFigure 2. Visual Plots of Impairment Scores, Heart Rate Changes, and Subjective Impairment Ratings per Condition
eFigure 3. Within-Individual Performance of the Prediction Model During Each Scan
eTable 5. Performance Metrics for Participants Impaired at Only One Post-THC Scan
eTable 6. Performance of All Models
eFigure 4. Impairment Scores from Resting-State fNIRS Across Demographic and Cannabis-Use Subgroups
eFigure 5. Channel-Wise ROC–AUC Scores for Resting-State and N-Back fNIRS Models
eReferences.

This supplemental material has been provided by the authors to give readers additional information about their work.

eMethods

Participants: Participants were excluded if they had allergies to sesame oil, were currently pregnant, were lactating, intended to become pregnant, or had schizophrenia, bipolar I disorder, diabetes, cirrhosis, renal failure, Hepatitis C, HIV, frequent migraines, significant head injuries, or any new, or unstable medical or psychiatric condition. Additionally, participants had to be able to complete the study procedures safely in the opinion of the investigators. Participants were recruited through community advertisements. Screening visits included urine THC-COOH concentration.[1] Participants were asked to abstain from consuming any intoxicating substances on the morning of their study visits. Urine was also tested for cannabinoids, opioids, cocaine, and amphetamines at the screening and each study visit. Participants who tested positive for any of these substances other than cannabinoids or demonstrated clinical signs of intoxication were rescheduled for a later date.

THC Dosing

To determine a clinically tailored dose of THC that would maximize the chance for intoxication while minimizing adverse events such as anxiety, panic, paranoia, and hemodynamic change, clinical staff took a careful history of cannabis product use, including age of onset and quantity/dose, frequency, and route of cannabinoid use over time, with particular attention to quantity and frequency of recent use, 0-100 rating of subjective intoxication with usual use, recent oral cannabinoid dosing, if any, with self-rated intoxication and adverse events. Study physicians (GP and AEE) determined during the screening visit the dronabinol dose most likely to be both well-tolerated and to produce intoxication, up to a maximum dose of 80 mg. Primary dosing considerations were degree of expected tolerance and prior adverse experiences with THC-containing products, particularly edibles. Doses were calculated for each participant individually based on their reported average cannabis frequency and demographic factors such as age and sex. Participants with a systolic blood pressure >130 and/or diastolic blood pressure >90 were given either 5mg (unlikely to produce further blood pressure increase) or their calculated dose based on the recommendation of the study physician. For participants with mild hypertension, study physicians determined dosing on a case-by-case basis. If the participant had no prior adverse cardiovascular reactions to cannabis and the calculated dose was judged to be safe, the individualized calculated dose was administered. If there was uncertainty regarding tolerance, prior adverse effects, or potential cardiovascular risk, a conservative fixed dose of 5 mg was used to minimize the chance of further blood pressure elevation. All dosing decisions were made by the study physician and documented in the source records.

Blinding Procedures. Participants were randomly assigned for order to receive a single oral dose of dronabinol, over-encapsulated synthetic THC capsules, and identical appearing placebo capsules on separate study visits at least 7 days apart. Study staff and participants were blinded to the contents of the containers. Allocation concealment was maintained by ensuring that the pharmacy, which had no role in participant interaction or data collection, was solely responsible for preparing and labeling the study drugs according to the randomization list. The random allocation sequence was generated using a computer-based permuted block randomization procedure with a 1:1 ratio for order of THC and placebo, stratified by sex, and was implemented using sequentially numbered, identically appearing opaque containers prepared by the investigational pharmacy.

Impairment Definition: As discussed in prior work,[2] there exists a lack of a universally

© 2026 Berchanskey M et al. *JAMA Network Open*.

accepted objective measure for impairment. It has been established that the eFST is associated with high false-positive rates,[3,4] and is prone to bias. Thus, we followed the two-step process developed previously to determine impairment.[2] In this process, post-THC participants are considered impaired at the time of the scan if (1) two clinical raters independently rated the participant as impaired, using all available data except fNIRS data, and (2) an algorithm combining physiological and psychological (heart rate and self- reported intoxication (DEQ)) classified the participant as impaired following study drug administration. The DEQ included five items ("Feel Drug," "Like Drug," "High," "Want more of what you received," and "Dislike Effects"), each rated on a 0–100 visual analog scale. Elevated "Feel Drug" and "High" ratings were the most consistent indicators of intoxication, though no single DEQ threshold was used as an absolute criterion. We note that participants could be classified as impaired even if they did not self-report intoxication, provided both raters judged that objective physiological and behavioral indicators were consistent with THC-induced impairment.

For this work, participants rated as impaired by both methods are viewed as impaired, serving as the ground truth for our models.

fNIRS Acquisition: Cortical hemodynamic data was collected using a continuous wave fNIRS system (NIRSport2, NIRx Inc., Germany) equipped with 8 sources emitting light at wavelengths of 760 and 850 nm, and 8 photodetectors. Each channel (a pair of source and detector) recorded changes in the relative concentrations of oxygenated hemoglobin (HbO) and deoxygenated hemoglobin (HbR) at a sampling rate of 7.81 Hz. NIRStar software from NIRx was utilized before each session to verify signal quality to ensure reliable data acquisition. All event markers associated with the NIRS data were displayed, recorded, and stored. The NIRS probe featured a configuration of eight light sources and seven detectors strategically positioned over each participant's prefrontal cortex (PFC) region. The placement followed the International 10-20 System,[5] with the central probe aligned with the Fpz site (**Fig 1**). The remaining probes were placed along the F5-Fp1-Fpz-Fp2-F6 axis. The cap's central point was positioned at the Cz vertex, equidistant from the nasion (Nz) and inion (Iz) and from the left and right preauricular points. Each source and detector probe pair has a distance ranging from 2.5 to 3 cm, with the midpoint of the source-detector distance defining the channel location for analysis. The fNIRS data were preprocessed using a standard pipeline,[6] including principal component analysis-based motion correction, filtering, and conversion from optical density to oxygenated and deoxygenated hemoglobin concentration measures. The oxyhemoglobin and deoxyhemoglobin time series from 20 channels (2x20 time series) per participant was defined as the input data to our model.

Evaluation of Data Quality:
Signal quality was evaluated using signal-to-noise ratio measures. Any dataset in which greater than 10% of channels demonstrated a signal-to-noise ratio (SNR) lower than 5 dB computed across the full recording, were considered poor quality and excluded as having suboptimal optical coupling.

We did not perform channel level elimination, as even channels with adequate physiological activity (e.g., cardiac fluctuations suggesting good scalp-optode contact) could exhibit variability due to motion, scalp curvature, or local hair density. Instead of selecting channels and potentially introducing a bias by disproportionately removing certain areas, we implemented a stricter a quality control criterion (>90% of channels) at the whole brain level

fNIRS Data Preprocessing

© 2026 Berchanskey M et al. *JAMA Network Open*.

Pre-processing fNIRS (functional Near-Infrared Spectroscopy) signals involves several key steps to clean and prepare the data for analysis. fNIRS data consists of intensity or optical density measurements of oxygenated (HbO) and deoxygenated (HbR) hemoglobin concentrations over time. Below is an outline of the typical steps used to pre-process fNIRS signals in Python, including handling noise, artifacts, and signal filtering. The processing was performed using the HOMER2 software.

Artifact Detection and Removal: Since artifacts are a common problem in fNIRS data (e.g., due to motion or muscle activity), we utilized Principal Component Analysis (PCA) to isolate and remove motion artifacts. Motion artifacts can also be reduced by high-pass filtering, which removes low-frequency noise from the signal.

Oxygenated and Deoxygenated Hemoglobin (HbO and HbR): We compute the concentrations of HbO and HbR by applying the differential path length factor (DPF) model, using the Modified Beer-Lambert Law.[7]

In addition, we perform a set of post-processing steps, as to ensure data integrity and consistency. These steps are as follows:

- **SD Geometry Update**: Updates the Source-Detector (SD) structure using measurements from a subject's forehead to ensure the geometry is accurate. It replaces any incorrect SD configuration with a more precise one.
- **SD Structure Check**: Verifies whether the SD structure matches the default NIRx Prefrontal 8x8 montage. If it doesn't, the script corrects it, assuming the default SD files are available and the raw data is in the specified folder structure.
- **Strip SS Information**: Removes short-separation (SS) information from '.nirs' files for compatibility with the N-back dataset. It moves the original SS files to a separate folder and creates non-SS versions in a new directory.
- **Trigger Fix and Data Crop**: Processes fNIRS resting data by cropping it to a 6-minute duration, starting from the first trigger. It also adjusts trigger information to correct issues like early, late, or multi-column triggers.
- **Signal Quality Estimation**: Assesses the quality of fNIRS signals by computing the Signal-to-Noise Ratio (SNR) for each channel. It helps determine the reliability of the collected data.

Beyond these steps, each model includes additional data processing steps before inserting the data into the model, which we denote as data transforms. These transforms could include some of the following:

- **Standard scaling:** Transforms the signal to have a mean of 0 and a standard deviation of 1, ensuring that the features are centered and have uniform variance.
- **Min-max scaling:** Transforms the signal to a fixed range of

$[-1, 1]$, by subtracting the minimum value and dividing by $max - min$, while preserving the relative relationships of the data.

- **Signal division into patches**: Involves splitting the signal into smaller, fixed-size segments that can overlap or not. This effectively increases the signal count, which allows for the creation of additional training examples. For our purposes, each segment of the original signal has the same label (impaired or not impaired).

Models

For our machine learning approach, we selected several state- of-the-art architectures for time series classification and modeling, enabling accurate impairment classification (**Figure S1)**. First, **Detach Rocket**[8] introduces Sequential Feature Detachment (SFD), a method for identifying and pruning redundant features in ROCKET- based[9] time series classification models, significantly reducing computational load while enhancing accuracy. Tested on the UCR archive, the resulting Detach Rocket models deliver better performance, increasing efficiency and interpretability without compromising accuracy. The method has been successfully applied to other brain signal data, including MEG and EEG.[10] **Tiny Time Mixers**

(Granite-TTM R2)[11] is a lightweight, fast model with fewer than 1M parameters, utilizing innovative techniques such as adaptive patching and dataset augmentation among others, to excel in multivariate time series forecasting and classification. Additionally, we experiment with **Granite PatchTST alongside Tiny Time Mixers and MiniRocket (Granite Combined)**.[8,11] This model combines four models: PatchTST,[12] PatchTST with time mixing, TTM, and MiniRocket[13] features. The model passes the input signal into each model, and each model extracts features from it. Then, the features are combined and are passed into a final module that projects the features into a class prediction score.

Participant and Label Division Between Folds

Since the division described above was done at the participant level, we note that the number of positive recordings per participant varied along with the total number of recordings. To ensure that all the recordings for each participant were in the same fold, we divided the participants among the 5 folds, ensuring that the positive- to-negative ratio in each fold is preserved as much as possible. This means that while the number of participants per fold may change, the overall count of positive and negative recordings per fold remained mostly constant. All segments/patches inherit the participant's fold, and no segment from a participant appeared in both train and test sets.

Machine Learning Time Series Modeling

Preliminaries

The time series data is typically structured as a sequence of observations over time. Let the time series data be denoted as:

$$x = \{x_1, x_2, \ldots, x_T\}$$

where x_t represents the feature set at time step t, and denotes the total number of time steps. For our purposes, the dimension consists of 20 Oxy channels, and 20 De-Oxy channels. The layout and location of these channels are illustrated in **Figure S6**. Each time series sequence is associated with a label, which our model must predict.
In general, we utilize a classification model $\mathrm{H}_{\{\mathrm{class}\}}$, for classifying the sequence into the required labels:

$$\hat{y} = \mathrm{H}_{\{\mathrm{class}\}}(\mathrm{x}) \in \mathrm{R}^2$$

where $\hat{y}_1$ and $\hat{y}_2$ are unnormalized predicted log-probabilities of positive and negative labels, respectively.

Loss Functions

For training our impairment classification models, we use multiple different losses, each for the appropriate architecture.

Cross Entropy Loss: Measures the difference between the classification distribution produced by the model and the ground truth. We compute the prediction distribution $\hat{z}$ for each scan using the softmax function:

$$\hat{z} = \frac{\mathrm{e}^{\hat{y}_1}}{\sum_{j=1}^{2} \mathrm{e}^{\hat{y}_j}}$$

© 2026 Berchanskey M et al. *JAMA Network Open*.

Thus, the loss for a single observation is computed as

$$L_{class} = -[\,y\log(\hat{z}) + (1 - y)\log(1 - \hat{z})]$$

where y is the true value.

The mean of these individual losses is taken as the total loss.

Ridge Regularization for Detach Rocket: To train the Detach Rocket model, we combine the mean squared loss, regularization, and feature selection. First, the ridge classifier loss function combines mean squared loss with an L2 regularization term, controlled by the λ parameter:

$$\underbrace{\sum_{i=1}^{N}\left(y_i - \theta_0 - \sum_{\{k \in S_t\}} x_{\{i,k\}}\,\theta_k\right)^2}_{Mean\ Squared\ error} + \underbrace{\lambda \sum_{\{k\}} \theta_k^2}_{\text{L2 regularization term}}$$

Here, N denotes the number of training examples, y_i is the class (-1 for not impaired and 1 for impaired), $x_{\{i,k\}}$ denotes the feature k of example i, and θ_k are the model coefficients for feature k and θ_0 serving as the bias term.

Then, feature selection uses iterative steps, with each step keeps a fixed percentage from the current active features.

In essence, in a given step t, we train a ridge classifier on the current active features at step t, denoted as S_t. The training is performed by solving

$$\hat{\theta}_t^{ridge} = \underset{\theta}{\operatorname{argmin}}\left(\sum_{i=1}^{N}\left(y_i - \theta_0 - \sum_{\{k \in S_t\}} x_{\{i,k\}}\,\theta_k\right)^2 + \lambda \sum_{\{k\}} \theta_k^2\right)$$

From this we obtain the coefficients $\hat{\theta}_t^{ridge} = \{\hat{\theta}_{\{t,k\}}\}$, with each $\hat{\theta}_{\{t,k\}}$ associated with feature k. Then, we rank the features by absolute value $|\hat{\theta}_{\{t,k\}}|$ and discard the least important features for the next step. During the process, the parameter λ is determined using cross-validation on the initial full model. For more details on the process, we refer the reader to the original paper [7].

Bootstrapped Estimation of Performance Variability and Significance Testing

To quantify the variability and uncertainty of classifier performance, we implemented a nonparametric bootstrap resampling procedure. For each model, we generated 1,000 bootstrap samples by sampling with replacement from the test predictions, preserving the original sample size. Within each bootstrap iteration, we recompute all performance metrics (F1 score, precision, recall (sensitivity), false positive rate (FPR), accuracy, and ROC–AUC) on the portion of each bootstrap sample.

© 2026 Berchanskey M et al. *JAMA Network Open*.

For each metric, we summarized variability by calculating the standard deviation of the bootstrap distribution and derived 95% confidence intervals using the percentile method (i.e., 2.5th and 97.5th percentiles of the bootstrap estimates). All bootstrapping procedures were implemented using custom Python scripts.

To formally test whether performance differences between machine-learning classifiers were statistically significant, we implemented a nonparametric bootstrap procedure. For each of 1,000 bootstrap iterations, we sampled with replacement to generate a bootstrap sample, and calculated performance.. This produced paired distributions of metric differences (Model A – Model B). For each metric (F1 score, precision, recall, ROC–AUC, false positive rate, and accuracy) we derived 95% confidence intervals from the 2.5th and 97.5th percentiles of the bootstrap difference distribution. A difference was considered significant if the p values are below 0.05.

Evaluation Metrics

Precision: Also known as Positive Predictive Value, is the proportion of true positive predictions among all positive predictions made by the model:

$$Precision = \frac{TP}{TP + FP}$$

where TP are the True Positives (correctly predicted positives), and FP are the False Positives (incorrectly predicted positives).

False Positive Rate: The proportion of false positive predictions among all negative predictions made by the model:

$$FPR = \frac{FP}{FP + TN}$$

where TN are the True Negatives (correctly predicted negatives), and FP are the False Positives (incorrectly predicted positives).

Recall: Also called Sensitivity or True Positive Rate is the proportion of true positive predictions among all actual positive instances:

$$Recall = \frac{TP}{TP + FN}$$

where FN are the False Negatives (actual positives incorrectly predicted as negatives)

F1-Score: The harmonic mean of Precision and Recall. It provides a balanced measure of these two metrics, especially useful when the dataset is imbalanced.

$$F1Score = 2\frac{Precsion * Recall}{Precision + Recall}$$

ROC-AUC (Receiver Operating Characteristic - Area Under Curve): Computed as the integral of the ROC curve, representing the probability that the model ranks a randomly chosen positive

© 2026 Berchanskey M et al. *JAMA Network Open*.

instance higher than a randomly chosen negative instance. In particular, the ROC Curve is a graphical plot that illustrates the diagnostic ability of a binary classifier as its discrimination threshold is varied. AUC computed for the area under the ROC curve, summarizing the trade-off between the True Positive Rate (TPR), which is also the recall, and the False Positive Rate (FPR), when computed across different thresholds

Precision-Recall Curve (PR Curve): A Precision-Recall curve is a graphical representation of the trade-off between precision and recall for different threshold values. Each point on the curve corresponds to a specific threshold. The Precision-Recall AUC is the area under the Precision-Recall curve. It summarizes the model's performance across all thresholds, with a higher value indicating better performance. It is computed using numerical integration methods such as the trapezoidal rule.

Adverse Events. Thirty-eight participants reported adverse events that were considered related to THC. The most common were anxiety (9 participants), vomiting (7 participants), nausea (6 participants), and dry mouth (5 participants). Dronabinol-induced tachycardia (HR>100) was observed in 41 participants and elevated blood pressure (systolic blood pressure (SBP) > 140) in 30 participants. All adverse events were considered mild to moderate and were transient. Asymptomatic severe hypertension (SBP > 180) was observed in 2 participants, correlating with peak drug effect.

© 2026 Berchanskey M et al. *JAMA Network Open*.

eTable 1: Baseline Characteristics

Measure	Subjects (n = 183)
Age (years); Mean (SD)	25.3 (6.3)
Sex; No. (%)	
-- Female	90 (49.2%)
-- Male	93 (50.8%)
Race; No. (%)[a]	
-- American Indian or Alaska Native	2 (1.1%)
-- Asian	12 (6.6%)
-- Black or African American	21 (11.5%)
-- White	126 (68.9%)
-- More than one race	14 (7.7%)
-- Unknown	8 (4.4%)
Ethnicity; No. (%)[a]	
-- Hispanic/Latino	36 (19.7%)
-- Not Hispanic/Latino	143 (78.1%)
-- Unknown	4 (2.2%)
Education (years); Mean (SD)	15.2 (2.1)
Cannabis use (days per week); Median (Q1, Q3)	6.5 (4, 7)
Urine CN-THCCOOH (ng/mL); Median (Q1, Q3)[b]	100.0 (33.5, 271.5)
CUDIT-R total score; Mean (SD)[c]	11.9 (5.3)
AUDIT total score; Mean (SD)[d]	6.0 (3.8)
Self-reported major depressive disorder; No. (%)	42 (23%)
Self-reported anxiety disorder; No. (%)	49 (26.8%)

[a] Race and ethnicity were assessed by participants' response to a fixed-category question.
[b] Creatinine-adjusted 11-nor-9-carboxy-tetrahydrocannabinol (CN-THCCOOH), the main secondary THC metabolite and a widely accepted cannabis biomarker.
[c] Cannabis Use Disorders Identification Test - Revised (CUDIT-R), an 8-item screening tool with scores from 0 to 32, with higher scores indicating greater severity of cannabis-related problems. A score of 8-11 indicates hazardous cannabis use and a score of 12 or more suggests a potential cannabis use disorder.
[d] Alcohol Use Disorders Identification Test (AUDIT), a 10-item screening tool with scores from 0 to 40, with higher scores indicating greater severity of alcohol-related problems. A score of 8-14 indicates likely harmful/hazardous drinking and a score of 15 or more suggests a potential alcohol use disorder.

© 2026 Berchanskey M et al. *JAMA Network Open*.

eTable 2: Participant Counts of THC Impairment Status and Scan Type

	Received THC and Impaired (n=84)		Received THC and Not Impaired		
Scan	Impaired at Both Scans	Impaired at One Scan	Impaired at Neither Scan	Received Placebo Only	Total
Resting	32	52	71	18	173
N-back	32	57	73	18	180

eTable 2. Distribution of participants by clinical impairment classification and scan type (Resting and N-back). A total of 173 participants had usable resting-state scans, and 180 had usable N-back scans. Note that 18 participants in each scan group received placebo only and were not administered THC. These counts reflect the final analytic sample used for model training and evaluation.

© 2026 Berchanskey M et al. *JAMA Network Open*.

eTable 3: Effects of Study Medication on THC Impairment

Measure	THC Impaired (n=84)	THC Not Impaired (n=71)	T-Test
THC dose (mg); Mean (SD)[a]	35 (11.7)	37.7 (17)	1.15 (120.9), p = 0.25
Subjective high (DEQ); Mean (SD)[b]	78 (18)	43.3 (30.7)	8.39 (108.7), p < 0.001
Heart rate change from baseline (bpm); Mean (SD)	31.5 (16.9)	14.1 (14.5)	6.91 (153), p < 0.001

[a] Dose of dronabinol, an FDA-approved synthetic THC.
[b] Drug Effects Questionnaire (DEQ) question assessing subjective high, ranging from 0 - 100.

eTable 3. THC dose, subjective drug effects, and physiological response between participants categorized as clinically Impaired vs not clearly clinically impaired following THC administration. The THC dose reflects the administered amount of dronabinol. Subjective high was assessed using the Drug Effects Questionnaire (DEQ), with scores ranging from 0 to 100. Heart rate change reflects the difference in beats per minute (bpm) from baseline following THC administration. Statistical comparisons were performed using independent samples t-tests. Significant differences were observed in subjective high and heart rate change, but not in THC dose, between the two groups.

© 2026 Berchanskey M et al. *JAMA Network Open*.

eTable 4: Statistical Comparison of fNIRS Resting-State and n-Back Models Across Classification Metrics

Metric	P-Value	CI Lower	CI Upper	Observed Difference	Standard Error
Precision	0.22	-0.04	0.10	0.02	0.0011
Recall	0.34	-0.09	0.04	-0.01	0.0012
F1 Score	0.46	-0.05	0.06	0.00	0.0010
Accuracy	0.31	-0.01	0.02	0.00	0.0002
False Positive Rate	0.15	-0.02	0.00	0.00	0.0002
ROC-AUC	0.12	-0.01	0.04	0.01	0.0004

eTable 4. Paired bootstrap significance tests comparing the Resting-State and n-Back machine learning models across six performance metrics. For each metric, the observed difference, 95% confidence interval, standard error, and p value were computed using 1,000 bootstrap resamples applied to the same set of instances. No statistically significant differences were detected between the two models, indicating comparable performance across all evaluated metrics.

© 2026 Berchanskey M et al. *JAMA Network Open*.

eTable 5: Performance Metrics for Participants Impaired at Only One Post-THC Scan

	Metric	Observed Value	CI Lower	CI Upper	Standard Error
Resting	Precision	0.63	0.50	0.76	0.021
	Recall	0.48	0.35	0.62	0.022
	F1 Score	0.55	0.42	0.65	0.019
	Accuracy	0.85	0.82	0.89	0.005
	False Positive Rate	0.06	0.03	1.0	0.005
	ROC-AUC	0.84	0.77	0.9	0.010

eTable 5. Statistical performance of the Detach Rocket resting-state model in the subset of participants who were clinically impaired at one, but not both, post-THC time points. Each metric is shown with its observed value and 95% confidence interval derived via bootstrap resampling, along with the corresponding standard error.

© 2026 Berchanskey M et al. *JAMA Network Open*.

eTable 6: Performance of All Models

	F1		Precision		Recall (sensitivity)		ROC-AUC		FPR		Accuracy	
	Resting	N-back	Resting	N-back	Resting	N-back	Resting	N-back	Resting	N-back	Resting	N-back
**Detach Rocket**	58.21	57.77	60.78	55.08	55.85	60.74	87.35	84.97	5.11	7.05	90.03	88.92
**Granite-TTM R2**	54.17	57.97	49.57	56.3	61.18	61.86	87.35	85.39	8.58	7.82	87.69	88.39
**Granite Combined**	55.87	57.11	61.46	57.86	52.13	59.04	85.96	84.53	4.6	7.56	90.05	88.27
**Only including scans conducted in clearly impaired participants**												
**Detach Rocket**	74.74	72.81	74.11	75.13	75.57	70.77	91.10	87.39	8.93	8.10	87.17	86.56
**Granite-TTM R2**	75.34	74.76	74.56	80.15	76.48	70.64	89.68	89.29	8.88	6.13	87.43	88.03
**Granite Combined**	72.87	69.84	74.17	73.90	71.98	67.08	88.80	87.95	8.62	8.49	86.48	85.24

eTable 6. Evaluation metrics for our primary model (Detach Rocket) and alternative models**.** The top set of rows display the results for models that include all participants, while the bottom set of rows present results when only clinically impaired participants are included in the training and validation.

© 2026 Berchanskey M et al. *JAMA Network Open*.

eFigure 1: An Overview of Model Architectures Used

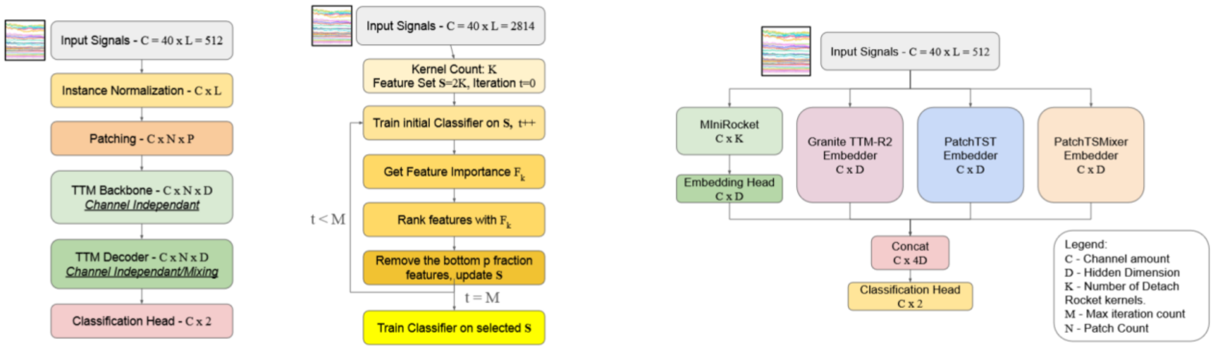

(a) Granite-TTM **(b) Detach Rocket** **(c) Granite Combined R2**

eFigure 1. An overview of various model architectures used. **Left (a):** The architecture of the Granite-TTM R2 model.[11] **Middle (b):** An illustration of the algorithm used to train the Detach Rocket [8] model. **Right (c):** The architecture of the Granite Combined model, which contains a concatenation of 4 sub models to produce the final classification score.

eFigure 2: Visual Plots of Impairment Scores, Heart Rate Changes, and Subjective Impairment Ratings per Condition

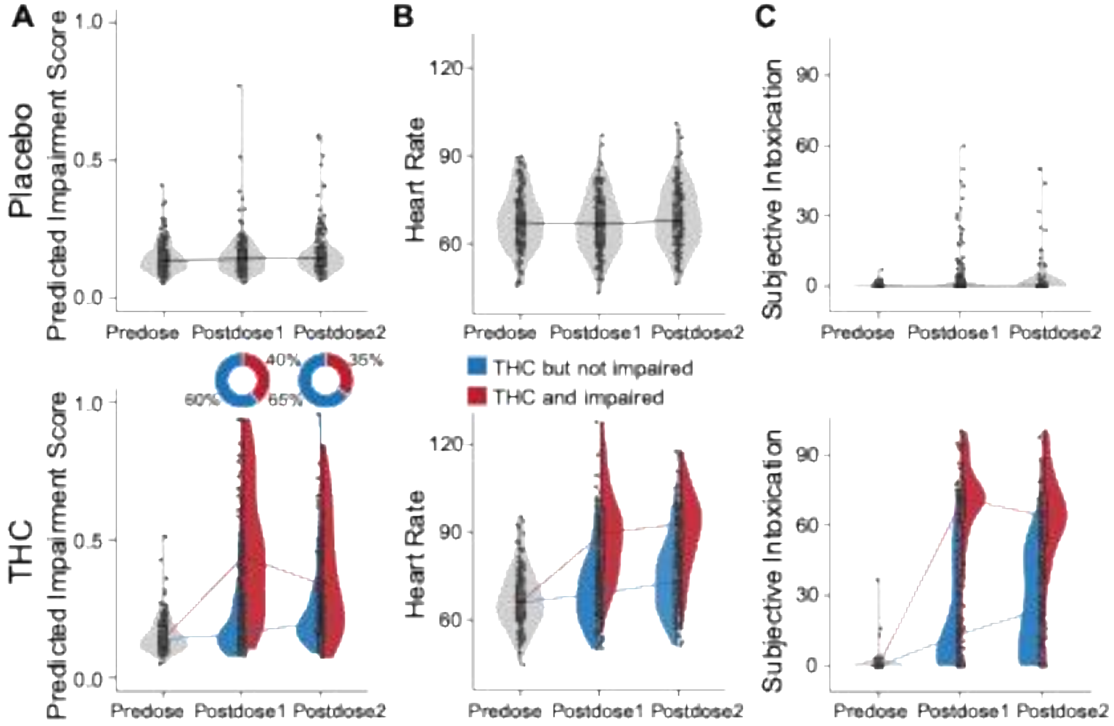

eFigure 2. Distributions of impairment-related features across metrics and timepoints. Violin plots show the variability and density of impairment-related metrics. The top row displays sample distributions during placebo. The bottom row displays distributions after THC administration, with red indicating participants classified as impaired and blue indicating those classified as not clearly impaired; lines connect repeated measures within individuals. Panel A depicts impairment scores, Panel B depicts heart rate, and Panel C depicts subjective intoxication ratings.

© 2026 Berchanskey M et al. *JAMA Network Open*.

eFigure 3. Within-Individual Performance of the Prediction Model During Each Scan

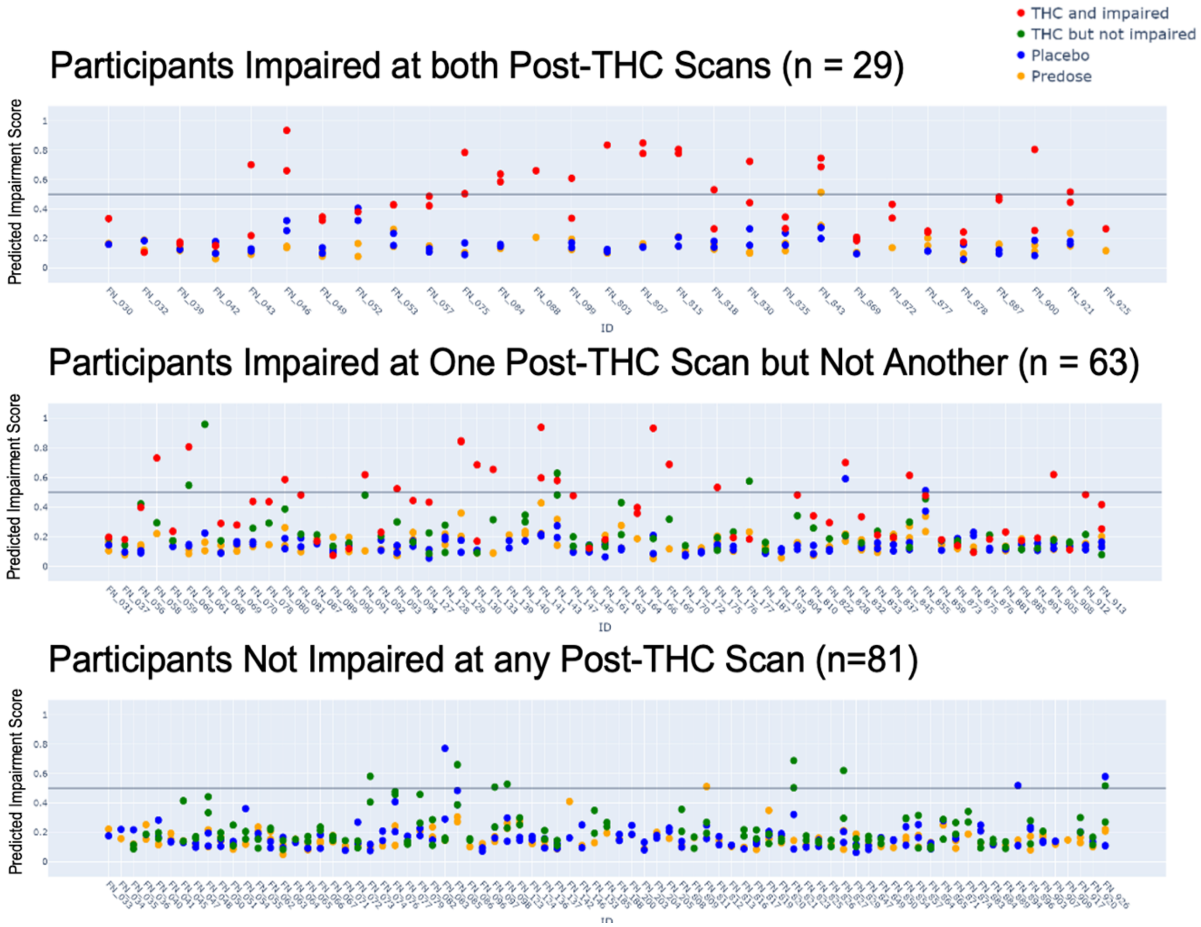

eFigure 3. Probabilistic predictions on the resting data for each scan of every participant during post-dose THC, post-dose placebo, and pre-dose scans. The y-axis of the graph represents the predicted impairment score. Color denotes scans in which participants received post-dose THC and become impaired (red), received post-dose THC but were not clinically impaired (green), received placebo (blue), and were assessed pre-dosing (orange). **(a)** Participants who were clearly impaired at all post-THC scans; **(b)** Participants who were impaired at one post-THC scan but not the other; **(c)** Participants who were never clearly impaired.

© 2026 Berchanskey M et al. *JAMA Network Open*.

eFigure 4. **Impairment Scores From Resting-State fNIRS Across Demographic and Cannabis-Use Subgroups**.

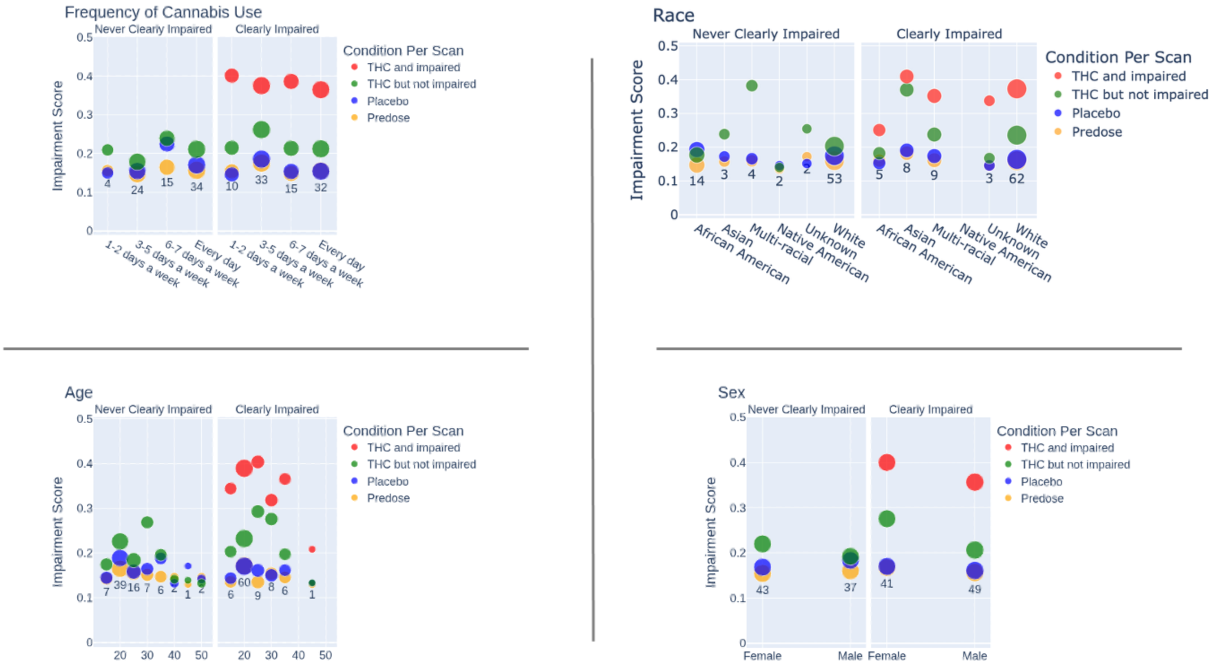

eFigure 4. Panels show impairment scores across all scans stratified by frequency of cannabis use (top left), race/ethnicity (top right), age (bottom left), and sex (bottom right). For each subgroup, impairment scores are displayed for scans in which participants received THC and were clinically impaired (red), received THC but were not impaired (green), received placebo (blue), or were assessed pre-dose (orange). Point size reflects the number of scans contributing to each category, and participant counts are displayed on the x-axis. Impairment differences were more pronounced among younger participants, while smaller differences in older age groups likely reflected reduced sample sizes. Race effects were influenced by signal-to-noise variability, with the smallest separation observed in African American participants, consistent with known fNIRS sensitivity to melanin-related light absorption. Both male and female participants showed higher impairment scores following THC when clinically impaired, compared with non-impaired scans. No clear pattern emerged across cannabis-use frequency groups.

© 2026 Berchanskey M et al. *JAMA Network Open*.

eFigure 5. Channel-Wise ROC–AUC Scores for Resting-State and N-Back fNIRS Models

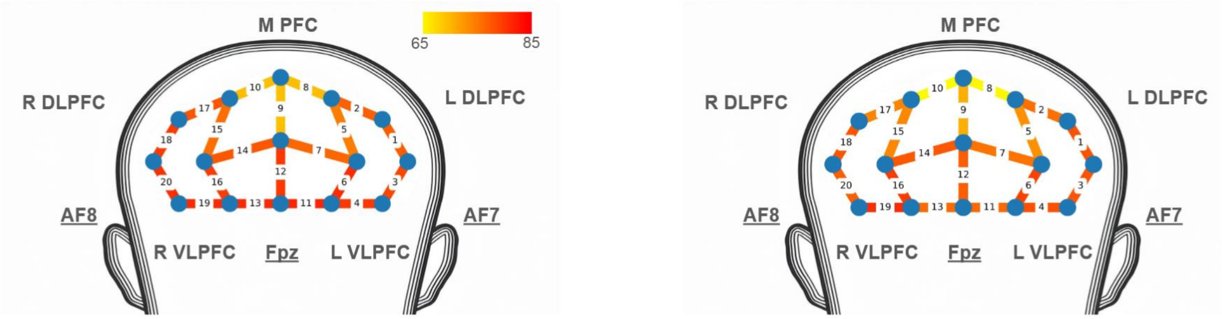

(a) Resting State

(b) N-back

eFigure 5. The mean ROC-AUC scores on the validation set for each channel of the Detach Rocket model, when running the model independently on each channel. Each line corresponds to a channel, with the color indicating the score of the model based on data from that channel. **Left:** resting state results; **Right:** N-back results.

© 2026 Berchanskey M et al. *JAMA Network Open*.

eReferences

1. Huestis MA, Cone EJ. Urinary excretion half-life of 11-nor-9-carboxy-delta9-tetrahydrocannabinol in humans. *Ther Drug Monit*. Oct 1998;20(5):570-6.
2. Gilman JM, Schmitt WA, Potter K, et al. Identification of Δ9-tetrahydrocannabinol (THC) impairment using functional brain imaging. *Neuropsychopharmacology*. Mar 2022;47(4):944-952. doi:10.1038/s41386-021-01259-0
3. Marcotte TD, Umlauf A, Grelotti DJ, et al. Evaluation of Field Sobriety Tests for Identifying Drivers Under the Influence of Cannabis: A Randomized Clinical Trial. *JAMA Psychiatry*. 2023;80(9):914-923. doi:10.1001/jamapsychiatry.2023.2345
4. Yoshizuka K PP, Upton G, Lopes I, Eric J Ip. Standardized Field Sobriety Test: False Positive Test Rate among Sober Subjects. . *J Forensic Toxicol Pharmacol* 2014;3(2)doi:doi:10.4172/2325-9841.1000120
5. Homan RW, Herman J, Purdy P. Cerebral location of international 10-20 system electrode placement. *Electroencephalogr Clin Neurophysiol*. Apr 1987;66(4):376-82. doi:10.1016/0013-4694(87)90206-9
6. Maki A, Yamashita Y, Ito Y, Watanabe E, Mayanagi Y, Koizumi H. Spatial and temporal analysis of human motor activity using noninvasive NIR topography. *Med Phys*. Dec 1995;22(12):1997-2005. doi:10.1118/1.597496
7. Delpy DT, Cope M, Zee Pvd, Arridge S, Wray S, Wyatt J. Estimation of optical pathlength through tissue from direct time of flight measurement. *Physics in Medicine & Biology*. 1988;33:1433 - 1442.
8. Uribarri G, Barone F, Ansuini A, Fransén E. Detach-rocket: sequential feature selection for time series classification with random convolutional kernels. *Data Mining and Knowledge Discovery*. 2024;38(6):3922-3947.
9. Dempster A, Petitjean F, Webb GI. ROCKET: exceptionally fast and accurate time series classification using random convolutional kernels. *Data Mining and Knowledge Discovery*. 2020/09/01 2020;34(5):1454-1495. doi:10.1007/s10618-020-00701-z
10. Solana A, Fransén E, Uribarri G. Classification of raw MEG/EEG data with detach-rocket ensemble: an improved rocket algorithm for multivariate time series analysis. Springer; 2024:96-114.
11. Ekambaram V, Jati A, Dayama P, et al. Tiny time mixers (ttms): Fast pre-trained models for enhanced zero/few-shot forecasting of multivariate time series. *Advances in Neural Information Processing Systems*. 2024;37:74147-74181.
12. Nie Y, Nguyen NH, Sinthong P, Kalagnanam J. A time series is worth 64 words: Long-term forecasting with transformers. *arXiv preprint arXiv:221114730*. 2022;
13. Dempster A, Schmidt DF, Webb GI. MiniRocket: A Very Fast (Almost) Deterministic Transform for Time Series Classification. *Proceedings of the 27th ACM SIGKDD Conference on Knowledge Discovery & Data Mining*. 2020;

© 2026 Berchanskey M et al. *JAMA Network Open*.
