# Supplementary material for: Detection of Δ9-Tetrahydrocannabinol Impairment Using Resting-State Functional Near-Infrared Spectroscopy: A Randomized Clinical Trial
Source: JAMA Netw Open. 2026 Jan 30;9(1):e2556647. doi: 10.1001/jamanetworkopen.2025.56647 (PMC12859723; doi:10.1001/jamanetworkopen.2025.56647)
Supplement: Supplement 3. — Data Sharing Statement [file jamanetwopen-e2556647-s003.pdf]

Data Sharing Statement

Berchansky. Detection of Δ9-Tetrahydrocannabinol Impairment Using Resting-State Functional Near-Infrared Spectroscopy. *JAMA Netw Open*. Published January 30, 2026. doi:10.1001/jamanetworkopen.2025.56647

Data
Additional Information: NCT03655717
Data available: Yes
Data types: Deidentified participant data, Data dictionary, Other (please specify)
Additional Information: analytic code
How to access data: The data that support the findings of this study are available from the corresponding author upon request. Data will include de-identified individual patient-level data, a data dictionary, and analytic code. Investigators proposing to use the data must execute a data use agreement with Massachusetts General Hospital and have approval from an Institutional Review Board (IRB), Independent Ethics Committee (IEC), or Research Ethics Board (REB), as applicable, before data is shared. Data will be available within three months of publication.
When available: With publication

Supporting Documents
Document types: None

Additional Information
Who can access the data: The data that support the findings of this study are available from the corresponding author upon request. Data will include de-identified individual patient-level data, a data dictionary, and analytic code. Investigators proposing to use the data must execute a data use agreement with Massachusetts General Hospital and have approval from an Institutional Review Board (IRB), Independent Ethics Committee (IEC), or Research Ethics Board (REB), as applicable, before data is shared. Data will be available within three months of publication.
Types of analyses: The data that support the findings of this study are available from the corresponding author upon request. Data will include de-identified individual patient-level data, a data dictionary, and analytic code. Investigators proposing to use the data must execute a data use agreement with Massachusetts General Hospital and have approval from an Institutional Review Board (IRB), Independent Ethics Committee (IEC), or Research Ethics Board (REB), as applicable, before data is shared. Data will be available within three months of publication.
Mechanisms of data availability: The data that support the findings of this study are available from the corresponding author upon request. Data will include de-identified individual patient-level data, a data dictionary, and analytic code. Investigators proposing to use the data must execute a data use agreement with Massachusetts General Hospital and have approval from an Institutional Review Board (IRB), Independent Ethics Committee (IEC), or Research Ethics Board (REB), as applicable, before data is shared. Data will be available within three months of publication.
